# Zwitterionic Electrolyte Additives Empowered Robust Zn–I_2_ Batteries Enduring a Low Temperature of −40 °C

**DOI:** 10.1002/advs.202518135

**Published:** 2025-11-06

**Authors:** Shuaibing Wang, Yulong Chen, Saddick Donkor, Zhanhu Guo, Gaopeng Wang, Yifan Li, Si Yu Zheng, Ben Bin Xu, Jintao Yang

**Affiliations:** ^1^ College of Materials Science & Engineering Zhejiang University of Technology Hangzhou 310014 P. R. China; ^2^ School of Engineering Physics and Mathematics Faculty of Science and Environment Northumbria University Newcastle upon Tyne NE1 8ST UK

**Keywords:** electrolyte additive, zinc‐iodine battery, Zn dendrites, zwitterionic

## Abstract

The shuttling of polyiodides in zinc‐iodine (Zn–I_2_) batteries causes severe active‐material loss and zinc‐anode corrosion, leading to poor cycling stability. In this work, zwitterionic pyrrole (ZiPy) is designed as a bifunctional additive to adsorb polyiodides and simultaneously stabilize the Zn anode. Experimental and theoretical results demonstrate that the incorporation of ZiPy regulates the interfacial pH, alters the solvation structure of Zn^2+^ ions, and promotes the preferential growth of zinc along the (002) crystal plane. Furthermore, the Zn–I_2_ battery incorporating ZiPy demonstrated superior cycling performance (completing 45 000 cycles at a high current density of 8 A g^−1^) and low‐temperature endurance, achieving a capacity retention of nearly 90.1% after 45 000 cycles at −40 °C. This work sheds light on the development of future high‐performance Zn–I_2_ batteries through zwitterionic electrolyte engineering.

## Graphical abstract

A zwitterionic pyrrole (ZiPy) additive is designed to simultaneously suppress polyiodide shuttling and stabilize the zinc anode in Zn–I_2_ batteries. ZiPy regulates interfacial pH, optimizes Zn^2+^ solvation, and promotes (002)‐oriented Zn deposition, enabling outstanding cycling stability over 45 000 cycles and excellent low‐temperature endurance, offering new insights for advanced Zn–I_2_ energy storage systems.

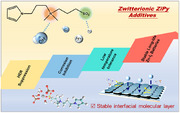

## 1 Introduction

Compared with lithium–ion batteries, aqueous zinc–ion batteries (AZIB) possess significant advantages, i.e., lower cost and better safety.^[^ ^1^, ^2^, ^3^, ^4^, ^5^ ^]^ The zinc‐iodine (Zn–I_2_) battery, a rising star of AZIB, attracts considerable interest due to the abundance of iodine resources and high theoretical specific capacity (211 mAh g^−1^ based on I_2_ + 2e^−^↔2I^−^ reaction) of the iodine‐based electrode.^[^ ^6^, ^7^ ^]^ However, the uneven migration of active species caused by polyiodide dissolution leads to electrode degradation and electrolyte decomposition.^[^ ^8^, ^9^, ^10^ ^]^ Moreover, polyiodides diffusing toward the zinc anode accelerate the occurrence of zinc dendrite, lower coulombic efficiency (CE), and deteriorate cycling performance.^[^ ^11^, ^12^ ^]^

To suppress the shuttle of multi‐iodide species, modification of the I_2_ cathode is a widely adopted strategy. Porous carbon hosts and polymer binders can effectively facilitate the redox conversion of iodine while mitigating the shuttling effect.^[^ ^13^, ^14^, ^15^ ^]^ Nevertheless, long‐term cycling stability in Zn–I_2_ batteries requires the durability of both the I_2_ cathode and the zinc anode.^[^ ^16^, ^17^, ^18^ ^]^ While the quasi‐solid and hydrogel electrolytes demonstrated their capabilities to promote uniform zinc deposition and suppress the shuttling effect, their complex and costly processes limit the practical application.^[^ ^19^, ^20^, ^21^ ^]^ From this perspective, electrolyte additives are attractive due to their facile preparation, good compatibility with existing industrial plants, and cost‐effectiveness.^[^ ^22^, ^23^ ^]^ In particular, organic macromolecular additives and ionic liquids can stabilize the iodine cathode through ionic interactions,^[^ ^24^ ^]^ but the associated increase in electrolyte viscosity hinders Zn^2+^ transfer kinetics, decreasing the rate performance of Zn–I_2_ batteries.^[^ ^25^, ^26^ ^]^ The challenge remains challenging to secure a unique additive that can sustain a good Zn^2+^ transfer kinetics toward a high‐performance of Zn–I_2_ batteries.

Zwitterions, with oppositely charged groups, are capable of regulating the flux of ions at the electrode/electrolyte interface and reducing side reactions on the electrode.^[^ ^27^, ^28^ ^]^ Specifically, zwitterionic electrolyte additives can induce the formation of a dynamic electrostatic shielding layer on the Zn anode, where the positively charged imidazole ring suppresses the zinc deposition tip effect, thereby enhancing the cycle life of batteries.^[^ ^29^ ^]^ The recent research found that the zwitterionic additive containing both pyridinium‐N cations and sulfonate anions can facilitate the desolvation of Zn^2+^ ions and suppress side reactions.^[^ ^30^ ^]^ However, the pyridine‐N structure with *π–π* conjugation exhibits limited pH buffering capacity.^[^ ^31^ ^]^ In contrast, the unshared electron pair from the nitrogen atom in the pyrrole participates in the conjugation of ring system, enabling strong adsorption interactions with the zinc anode, supporting the dynamic pH balance.^[^ ^32^ ^]^ The nitrogen‐containing groups with electron‐withdrawing capabilities, such as quaternary ammonium cations, can effectively adsorb polyiodides, reduce the vapor pressure of iodide‐containing species, and simultaneously inhibit the 2D diffusion of Zn^2+^.^[^ ^33^, ^34^ ^]^ Therefore, zwitterionic molecules containing pyrrolic‐N structures are promising electrolyte additives for Zn–I_2_ batteries, which remain yet to be fully explored.

Herein, we developed a bifunctional electrolyte additive of zwitterionic and pyrrolic‐N motifs (denoted as ZiPy). The pyrrolic‐N and quaternary ammonium cation moieties facilitate the formation of a molecular interface layer on the surface of the zinc anode, contributing to pH stability at the electrode‐electrolyte interface and effective adsorption of polyiodides. The incorporation of sulfonic acid anions further guides the migration of Zn^2+^, while the zwitterionic structure enhances ion‐dipole interactions in high‐concentration salting‐out solutions (Zn(ClO_4_)_2_ solution). The ZiPy can effectively inhibit the hydrogen evolution reaction (HER) on the Zn anode surface while lowering the reaction's activation energy. The incorporation of ZiPy reconstructs the Zn^2+^ solvation structure and the hydrogen bonding network within the electrolyte, thereby extending the cycle life of the cell. As such, Zn–I_2_ batteries based on ZiPy demonstrated exceptional cycling stability and impressive endurance to low temperature (‐40 °C).

## 2 Results and Discussion

**Figure 1:**
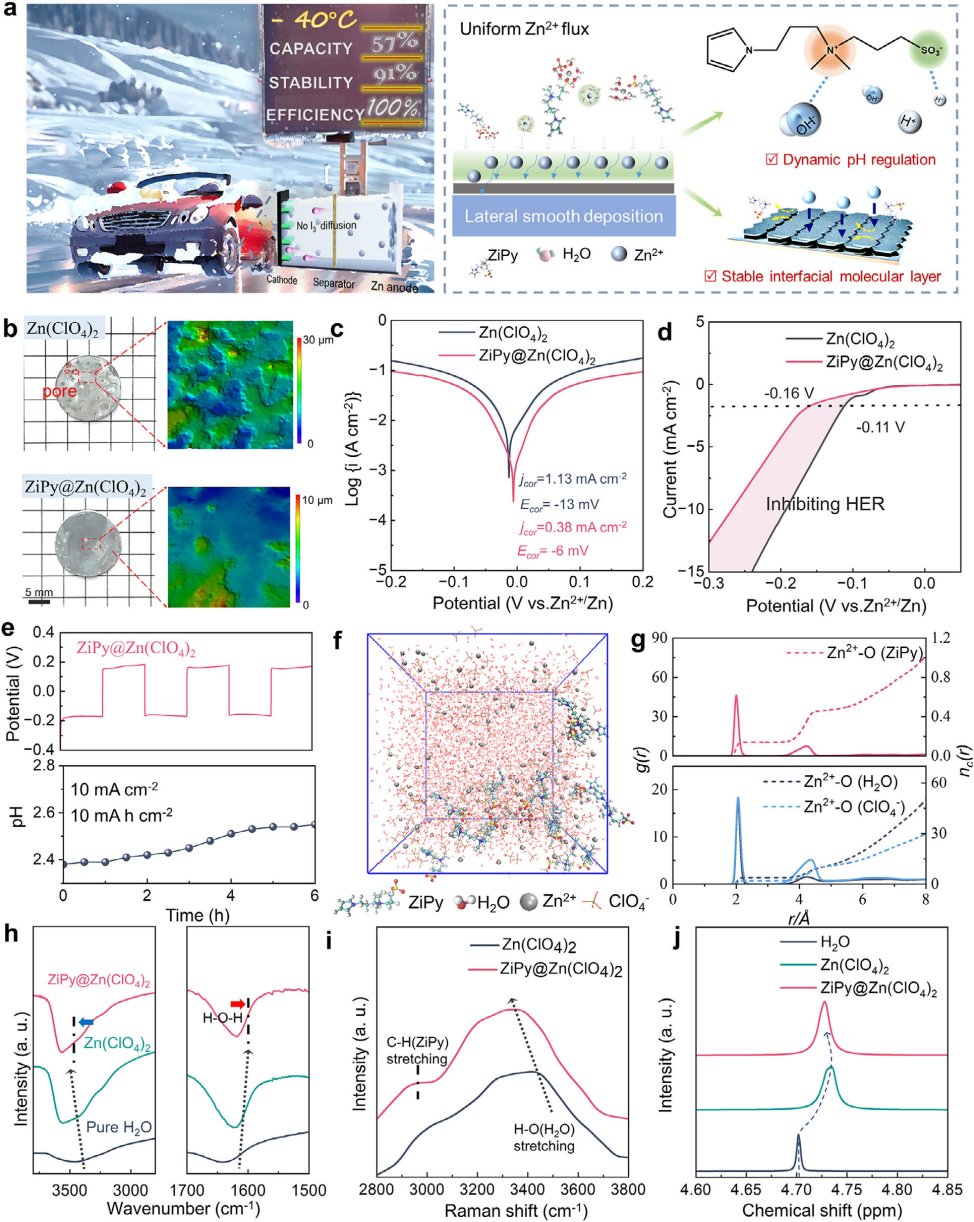
a) Schematic diagram of the beneficial effects of using ZiPy additive and corresponding chemical structure. b) Optical and CLSM images of zinc foils after being immersed in different electrolytes for 7 days. c) Tafel plots at varied electrolytes. d) The LSV curves of Zn||Zn symmetric batteries to evaluate the HER. e) pH monitoring of the ZiPy@Zn(ClO_4_)_2_ electrolytes of Zn//Zn symmetric cells cycling at 10 mA cm^−2^. f) 3D snapshot of the ZiPy‐containing additive system and the solvation structure of [ZiPy‐Zn(H_2_O)_5_]^2+^ obtained from MD simulations. g) RDF (g(*r*), left axis) and coordination number (*n*_c_(*r*), right axis) of O atoms in ClO_4_^−^, H_2_O, and ZiPy around a Zn^2+^ obtained from MD simulation. h) The IR, i) Raman and j) NMR spectra of different electrolytes.

**Figures** **1**a and S1 (Supporting Information) show the chemical structure and synthesis route of zwitterionic pyrrole (ZiPy). In the pristine Zn(ClO_4_)_2_ electrolyte, the nonuniform Zn deposition and uncontrolled shuttling of iodides exacerbate anode corrosion and promote dendrite formation (Figure S2, Supporting Information). By contrast, the addition of ZiPy effectively suppresses the formation of iodides and promotes uniform zinc deposition. To evaluate the effect of ZiPy, Zn||Zn symmetric batteries were tested to assess the impact of ZiPy additive, with using 5 m Zn(ClO_4_)_2_+0.24 mm ZiPy (ZiPy@Zn(ClO_4_)_2_) as the optimal electrolyte (Figure S3, Supporting Information). The preservation effect of ZiPy additive on the zinc anode can be observed through the self‐corrosion behavior of zinc foil (Figure 1b). Being immersed in pure Zn(ClO_4_)_2_ electrolyte for 7 days, the zinc foil's surface becomes rough, signaling severe self‐corrosion. After adding ZiPy, the confocal laser scanning microscope (CLSM) images clearly show a smoother zinc foil surface. For the zinc foils immersed in pure Zn(ClO_4_)_2_ electrolyte without ZiPy, the X‐ray diffraction (XRD) patterns evidence the generation of byproducts (Figure S4, Supporting Information), which doesn't appear to be a case in the sample with ZiPy. Figure S5 (Supporting Information) shows the wettability of different electrolytes on the zinc anode. The introduction of ZiPy decreased the contact angle from 86.3° to 47.2°, suggesting that ZiPy lowers the interfacial free energy of the anode. In the Tafel analyses, the corrosion current density of the Zn electrode in ZiPy@Zn(ClO_4_)_2_ is 0.38 mA cm^−2^ and the corrosion potential is ‐6 mV. However, the corrosion current density in the pure electrolyte is 1.13 mA cm^−2^ and the corrosion potential is ‐13 mV (Figure 1c), clearly proving the anti‐corrosion effect for ZiPy additive.

In the linear sweep voltammetry (LSV) tests (Figures 1d; S6, Supporting Information), the hydrogen evolution reaction (HER) inhibition effect of ZiPy is discovered with the explicit current window and pH changes in electrolyte during symmetric cell cycling (Figures 1e; S7, Supporting Information).^[^ ^35^ ^]^ The pH value of Zn(ClO_4_)_2_ electrolyte increase from 2.25 to 2.98 after cycling at 10 mA cm^−2^ for 6 h, whereas the value for electrolyte containing ZiPy seems be ignorable. A quick observation of post‐cycling electrode (Figure S7b, Supporting Information) suggests a uniform zinc deposition with ZiPy, as a result of a tender Zn anode corrosion driven by the electrochemical reaction kinetics.^[^ ^6^ ^]^

We next performed molecular dynamics (MD) simulations to understand the effect of ZiPy additives on the solvation structure of Zn^2+^. In the pure Zn(ClO_4_)_2_ electrolyte, Zn^2+^ primarily forms a solvation sheath with H_2_O and ClO_4_^−^, leading to a higher proportion of free water (Figure S8, Supporting Information). Interestingly, the system with ZiPy displays coordinated water molecule (Figures 1f; S9, Supporting Information), leading to the formation of a [ZiPy‐Zn(H_2_O)_5_]^2+^ solvation shell,^[^ ^36^ ^]^ with the first peak of radial distribution functions (RDFs) for O atoms in H_2_O, ClO_4_^−^, and ZiPy molecules around Zn^2+^ appears at ≈2 Å. The calculated coordination numbers (the molecules within the first peak of RDFs) of H_2_O and ZiPy around a Zn^2+^ are 3.85 and 0.15, respectively (Figure 1g).

The ZiPy effect on desolvation is also assessed by Fourier‐transform infrared spectroscopy (FTIR), Raman spectroscopy and nuclear magnetic resonance (NMR) tests. By adding Zn(ClO_4_)_2_ and ZiPy, the O─H bending band shifts to 1621.8 and 1617.9 cm^−1^, respectively. The displacement of O─H bond at 3560 cm^−1^ further demonstrates that the ClO_4_^−^ anion and ZiPy additives reconstruct the H‐bond network in the solvent (Figure 1h).^[^ ^37^ ^]^ The C─H stretching vibration at 2860–3010 cm^−1^ proves the addition of ZiPy, and the shifts for O─H stretching vibration at 3200–3600 cm^−1^ indicate its impact on hydrogen bonding environment (Figure 1i).^[^ ^38^, ^39^ ^]^ Calculations based on the deconvoluted peak areas showed that the introduction of ZiPy increased the proportion of strong and medium hydrogen bonds, whereas the fraction of weak hydrogen bonds decreased. These findings indicate that ZiPy effectively reconstructed the hydrogen‐bond network within the electrolyte (Figure S10, Supporting Information). In Figure 1j, the NMR result indicated the ^2^H peak of pure D_2_O is located at 4.70 ppm, the peak shifts to 4.734 ppm after adding Zn(ClO_4_)_2_, as a result of the strong coordination between Zn^2+^ and D_2_O which reduces the presence of free water molecules in the Zn(ClO_4_)_2_ solution. The addition of ZiPy causes the ^2^H peak shift to 4.727 ppm, suggesting that ZiPy participates in the desolvation of zinc ions, thereby increasing the number of free water molecules.^[^ ^40^, ^41^ ^]^

**Figure 2:**
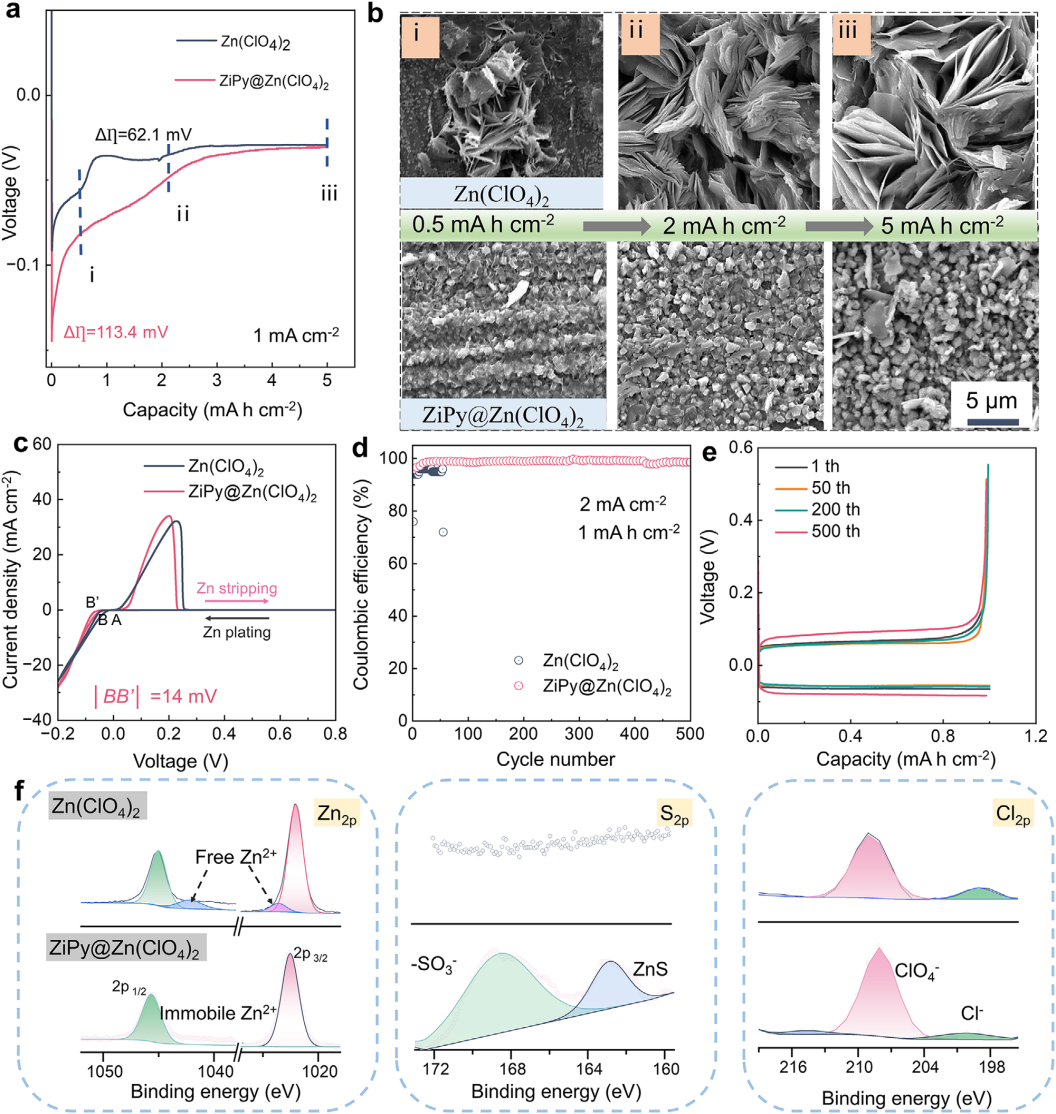
a) The first plating voltage profiles of Zn||Cu battery at 1 mA cm^−2^ and 5 mAh cm^−2^, and corresponding b) SEM images of at different areal capacities. c) CV curves of Zn nucleation process on Cu foil. d) CE changes of Zn||Cu half‐cell at 2 mA cm^−2^, and e) voltage profiles at different cycles. f) XPS results of Zn||Cu half‐cell cycled at varied electrolytes for 20 times.

Chronoamperometric analysis was then performed to examine nucleation behavior. In **Figure** **2**a, the nucleation overpotential for ZiPy@Zn(ClO_4_)_2_ electrolyte was significantly higher than that of pure Zn(ClO_4_)_2_ electrolyte, with a difference(Δη) of 113.4 mV, between the platform potential and nucleation overpotential. This higher nucleation overpotential is likely to improve the deposition of zinc.^[^ ^42^ ^]^ The deposition morphology of zinc on Cu foil was observed by scanning electron microscopy (SEM). For the ZiPy@Zn(ClO_4_)_2_ electrolyte, a uniform and dense layer of zinc deposition was formed (Figure 2b), whereas a coarse flake pattern was observed in the pure Zn(ClO_4_)_2_ electrolyte with dramatically reduced apparent surface area and developed spatially with even less surface area as the area capacity increases. The electrochemical performances for the above system were justified in Figure 2c with the first CV curve of zinc deposition on the bare Cu foil. The electrolyte containing ZiPy exhibits a larger nucleation overpotential, which reduces the nucleation radius of zinc ions on the anode surface and leads to the uniform and fine nuclei. The plating/stripping behavior of Zinc was assessed by long‐cycle testing of Zn||Cu asymmetric battery at 2 mA cm^−2^ and 1 mAh cm^−2^ (Figure 2d). The CE of the asymmetric battery with ZiPy@Zn(ClO_4_)_2_ electrolyte remains stable at over 98.5% for 500 cycles, while the Zn||Cu battery with pure Zn(ClO_4_)_2_ electrolyte fails quickly after 50 cycles. The voltage curves at different cycle numbers in Figure 2e verify a highly reversible Zn plating/stripping behavior enabled by ZiPy. It is worth noting that the ZiPy@Zn(ClO_4_)_2_ electrolyte demonstrates excellent stability, effectively cycling for 900 cycles at 5 mA cm^−2^ and 1 mAh cm^−2^ (Figure S11, Supporting Information).

The X‐ray photoelectron spectroscopy (XPS) was employed to analyze the deposition dynamics of zinc in the Zn||Cu half‐cell (Figure 2f). After 20 cycles, the Zn 2p spectrum in the Zn(ClO_4_)_2_ electrolyte can be decomposed into four peaks, with new peaks appearing at 1023.6 and 1042.2 eV, corresponding to the formation of Zn─O, indicating that free Zn^2+^ is coordinated with H_2_O molecules on the crystal surface. In the ZiPy@ Zn(ClO_4_)_2_ electrolyte, two prominent peaks corresponding to Zn 2p3/2 and Zn 2p1/2 are observed, with a peak shift of +0.53 eV, confirming a change in the chemical environment after ZiPy addition. Compared to the Zn(ClO_4_)_2_ electrolyte, the S2p peaks in ZiPy@Zn(ClO_4_)_2_ electrolyte appear at 168.4 eV (‐SO_3_^−^) and 162.9 eV (ZnS). The S2p peaks are attributed to ZiPy‐derived sulfonate groups, and the Cl 2p3/2 peak at 199.1 eV indicates Zn─Cl─O species from partial ClO_4_^−^ decomposition. These signals imply the formation of a dynamic interfacial environment, in which electrolyte species coordinate at the Zn surface. This dynamic intermediate layer mitigates side reactions while allowing Zn^2^⁺ transport.^[^ ^43^ ^]^

**Figure 3:**
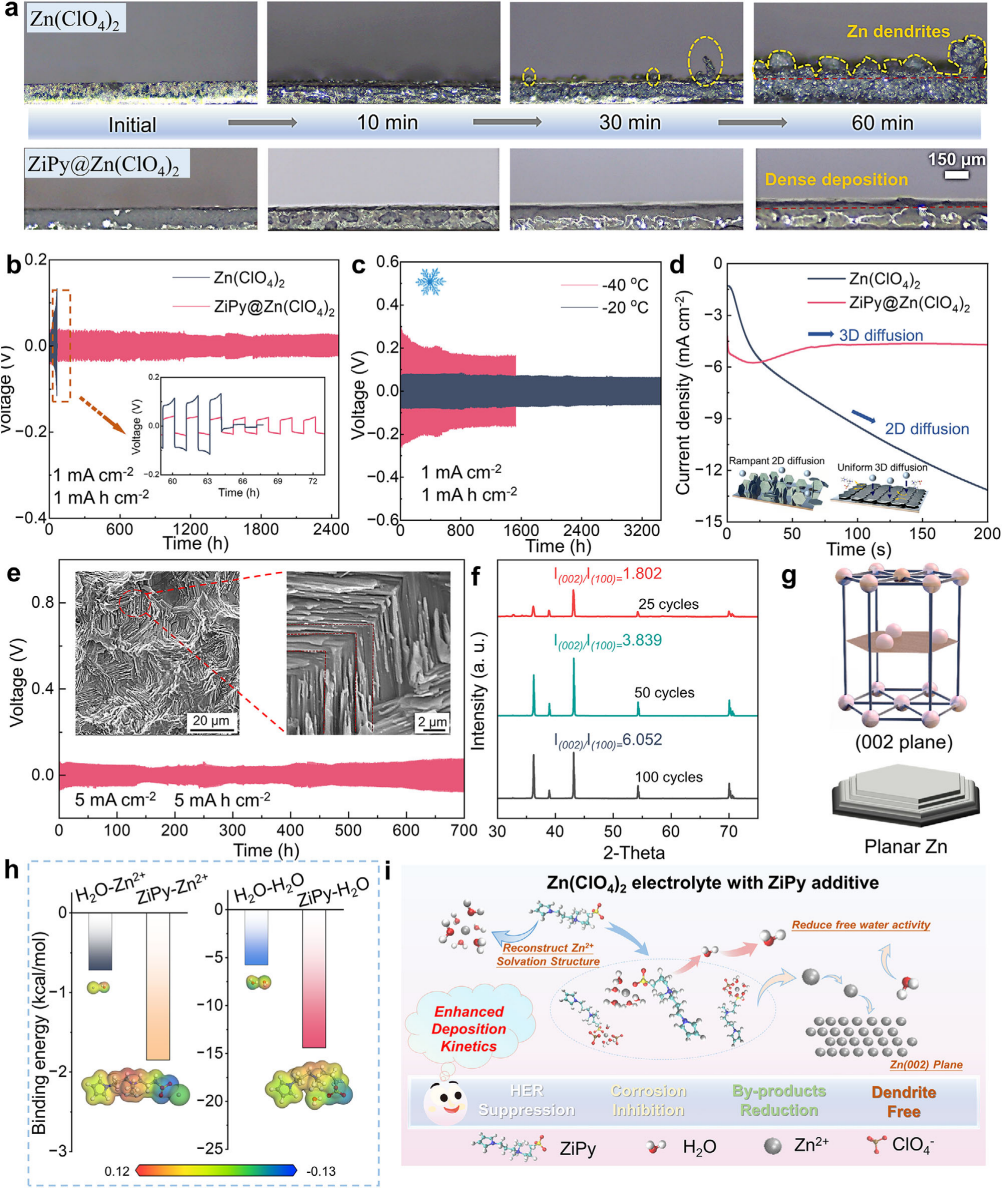
a) Optical microscopy images of in situ Zn deposition recorded in different electrolytes (10 mA cm^−2^). Cyclic deposition curves of zinc symmetric cells under b) 1 mA cm^−2^ and 1 mAh cm^−2^, and c) different temperatures. d) CA curves of different electrolytes at −100 mV overpotential. Cyclic deposition curves of zinc symmetric cells under e) 5 mA cm^−2^ and 5 mAh cm^−2^, the inset SEM images shows the deposition morphology after 50 cycles. f) XRD patterns of zinc foils after different cycles in ZiPy@Zn(ClO_4_)_2_ electrolyte. g) Schematic diagram of the Zn 002 crystal structure. h) ESP and binding energy of H_2_O‐Zn^2+^ and ZiPy‐Zn^2+^ from DFT calculations. i) Diagram explaining the improved zinc deposition behavior after adding ZiPy.

Under a high current density of 10 mA cm^−2^, dendrites occur on the Zn surface in the pure Zn(ClO_4_)_2_ electrolyte within 10 min (**Figure** **3**a), but the Zn surface of ZiPy@Zn(ClO_4_)_2_ electrolyte remains smooth throughout the electroplating process (Movie S1, Supporting Information). The effectiveness of ZiPy was further evaluated by long‐term cycling of symmetric Zn||Zn cells. Under 1 mA cm^−2^/1 mAh cm^−2^, the addition of pyrrole to the Zn(ClO_4_)_2_ electrolyte extends the cycle life of symmetric battery with a quintuple life‐time (from 63 to ≈324 h, Figure S12, Supporting Information). The addition of ZiPy increases the cycle life to 2450 h, an unprecedented 39‐fold improvement compared to the unmodified electrolyte, due to the gain effects of pyrrole‐N and zwitterionic structures (Figure 3b). More importantly, the Zn||Zn symmetrical battery can stably cycle for 3450 h even at a low temperature of −20 °C, and can still cycle for 1600 h at −40 °C, showing excellent low‐temperature resistance (Figure 3c). The chronoamperometry (CA) results (overpotential of −100 mV) indicate a continuous increase in current density for the pure Zn(ClO_4_)_2_ electrolyte, as a result of uncontrollable dendritic Zn growth caused by 2D Zn^2+^ ions diffusion. However, the ZiPy@Zn(ClO_4_)_2_ electrolyte exhibits a long‐term 3D diffusion process following to a brief period of 2D diffusion (≈50 s), enabled by the auxiliary deposition effect from ZiPy additive, which promotes the formation of a smooth and dense Zn deposition layer on the electrode surface (Figure 3d).^[^ ^44^ ^]^ Besides, as presented in Figure S13 (Supporting Information), the Zn^2+^ transference number of the ZiPy@Zn(ClO_4_)_2_ electrolyte reaches 0.45, markedly higher than that of the pristine Zn(ClO_4_)_2_ electrolyte (0.26), which facilitates faster Zn^2+^ ion transport for the batteries.

Under 5 mA cm^−2^/5 mAh cm^−2^, the cycle life of ZiPy@Zn symmetrical cell achieves ≈700 h (Figure 3e). In Figure S14 (Supporting Information), the rate cycling performance shows a stable voltage curve for ZiPy@Zn(ClO_4_)_2_ battery across varying current densities, while the data of pure Zn(ClO_4_)_2_ electrolyte exhibits significant fluctuations and short circuits at a current density of 5 mA cm^−2^. The XRD results reveal that the presence of sulfonic acid groups (‐SO_3_^−^) in ZiPy effectively induces the deposition of “002” crystal planes (Figure 3f,g).^[^ ^45^ ^]^ The formation of a hexagonal “002” crystal plane stacking structure was observed (Figure 3e), confirming the benefits from ZiPy additive on Zn deposition.

Interestingly, the DFT results in Figure 3h show that the addition of ZiPy is more conducive to binding with Zn^2+^. When Zn^2+^ is close to the sulfonic acid base, the increase in the potential value of ZiPy molecule proves the enhancement of electrostatic interaction, which is conducive to the formation of a more stable binding in a solution environment.^[^ ^46^, ^47^ ^]^ Thus, by modifying the Zn^2+^ solvation structure, ZiPy effectively inhibits zinc anode corrosion in ZiPy@Zn(ClO_4_)_2_ electrolytes and mitigates the risks of dendrite growth and the hydrogen evolution reaction during cycling. The zwitterionic structure of ZiPy also regulates the uniform deposition of Zn^2+^ along the “002” crystal plane, thereby extending the cycle life of symmetric batteries (Figure 3i).

**Figure 4:**
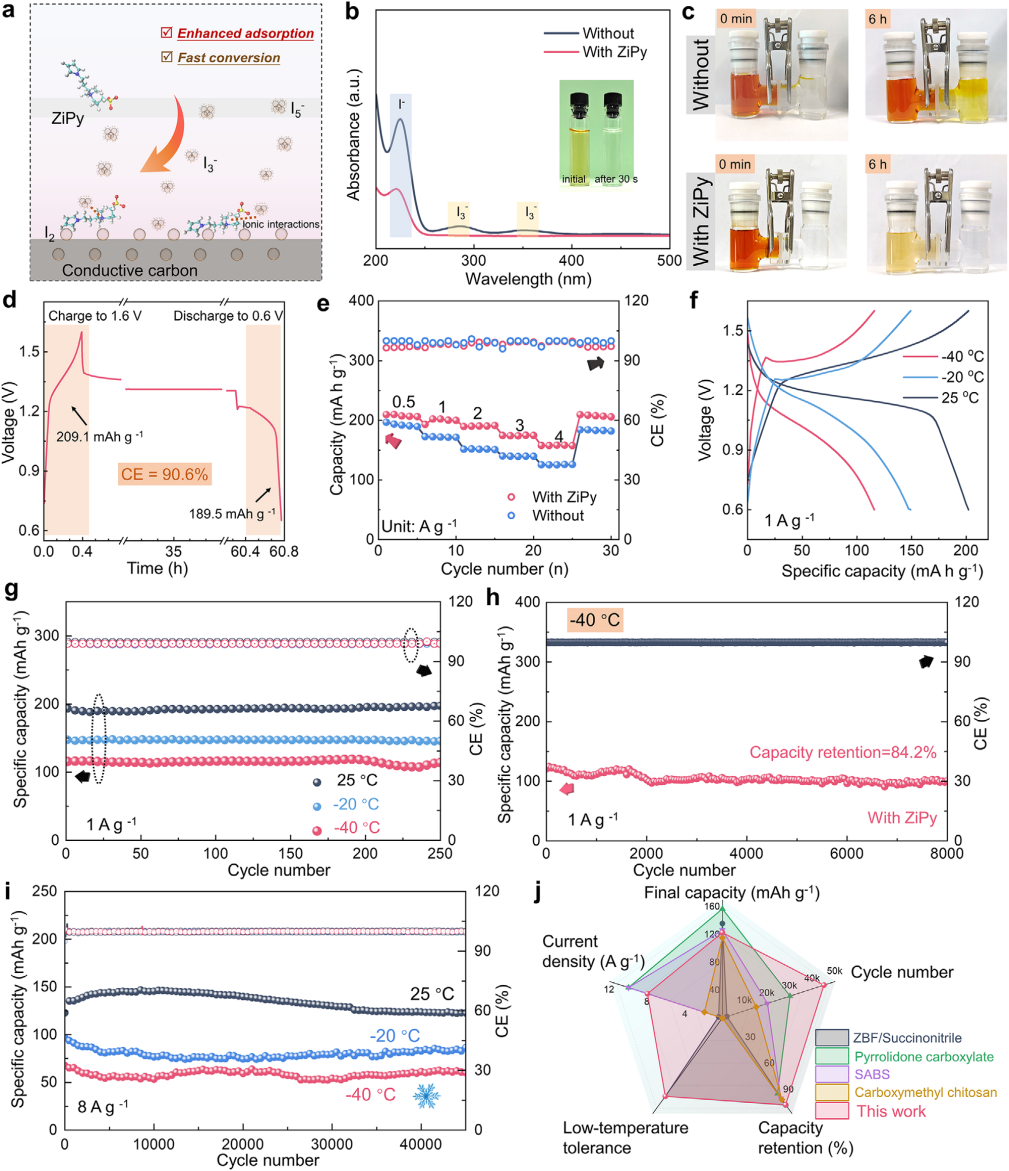
a) The illustrated mechanism of ZiPy working on the cathode. b) UV spectra and images of I_2_/Zn(ClO_4_)_2_ solution before and after adding ZiPy. c) Visualization of I_3_^−^ shuttling effect in H‐type cells with and without ZiPy. d) Capacity retention of Zn–I_2_ battery in ZiPy@Zn(ClO_4_)_2_ electrolyte after 60 h. e) The rate performance of Zn–I_2_ battery in varied electrolytes. f) GCD curves of Zn–I_2_ battery at 25, −20 and −40 °C. g) Capacity retention and CE of Zn–I_2_ full battery at different temperatures. h) Capacity retention and CE of Zn–I_2_ full battery at −40 °C. i) Capacity retention and CE of Zn–I_2_ full battery at 8 A g^−1^ at different temperatures. j) The performance comparison with previously reported Zn–I_2_ batteries.

We next assemble and characterize the ZiPy additives based full Zn–I_2_ battery, by utilizing activated carbon‐loaded iodine as the positive electrode and an iodine loading of 68 wt.% (Figure S15, Supporting Information). The working principle is hypothesized in **Figure** **4**a, the ionic interactions and chemical adsorption between iodide and ZiPy effectively suppress the shuttle effect, while the rapid charge transfer occurred at the solid–liquid interface enhances the electrochemical performance of the battery. As shown in Figure S16 (Supporting Information), the adsorption energy calculated by simulation shows that polyiodide prefers to bind to ZiPy rather than Zn^2+^, which can effectively reduce the reaction between polyiodide and the zinc anode. By adding ZiPy, the solution transitions from yellow to transparent (inset of Figure 4b) within 30 s, and the observed decrease in ultraviolet peak intensities at 288 and 350 nm (Figure 4b) confirms the reduction in polyiodide concentration. In Figure 4c, an experimental setup to assess the shuttle effect of polyiodides is built consisting of the left section of an H‐type glass tube to contain an aqueous solution of iodine and ZnI_2_, the middle section to comprise a glass fiber separator, and the right section to hold a colorless Zn(ClO_4_)_2_ solution. Without ZiPy additives, the right side of glass tube exhibits noticeable discoloration after 6 h due to the shuttle effect of polyiodides; however, the interaction between ZiPy and polyiodides mitigates this phenomenon, confirming the strong trapping capability of the additive.

Electrochemical analyses further highlight the benefits of ZiPy. The cyclic voltammetry (CV) curves (Figure S17, Supporting Information) present a typical two‐electron reaction and the presence of reversible redox peaks indicate the exceptional stability of Zn–I_2_ battery. After charging to 1.6 V and resting for 60 h, the battery containing ZiPy exhibit a capacity retention of up to 90.6% (Figures 4d; S18, Supporting Information), which is significantly higher than that of pure Zn(ClO_4_)_2_ electrolyte (78.2%). The rate performance of full cells is summarized in Figures 4e and S19 (Supporting Information). At a current density of 4 A g^−1^, the Zn–I_2_ battery containing ZiPy electrolyte maintains a capacity of 160 mAh g^−1^, showcasing its excellent performance.

Owing to the excellent anti‐freezing performance of perchlorate, the assembled Zn–I_2_ battery is expected to operate well at low temperatures. Figure S20 (Supporting Information) presents the Differential Scanning Calorimetry (DSC) results before and after adding ZiPy. No significant freezing is observed in either electrolyte at the ultra‐low temperature of −40 °C. We measured the temperature‐dependent viscosity and ionic conductivity in Figure S21 (Supporting Information). At different temperatures, the ZiPy‐containing electrolyte exhibited higher conductivity and lower viscosity compared to the Zn(ClO_4_)_2_ electrolyte. The reduced viscosity facilitates electrolyte penetration into the electrodes and improves ionic conductivity.^[^ ^48^ ^]^ At ‐20 °C (Figure 4f), the ZiPy based Zn–I_2_ battery retains a capacity of 149.2 mAh g^−1^ at a current density of 1 A g^−1^ and maintains 115.9 mAh g^−1^ at −40 °C, demonstrates its superior low temperature resistance. Although the capacity varies with temperature, the battery exhibits excellent cycling stability under all tested conditions (Figure 4g). Furthermore, the battery with the ZiPy additive maintains a capacity retention of up to 84.8% after 5000 cycles at −20 °C, whereas the control sample undergoes significant performance degradation after only 500 cycles even at room‐temperature (Figure S22, Supporting Information). The ZiPy additive explicitly inhibits the presence of polyiodide compounds and enhances capacity retention. Even at an I_2_ loading of 13.3 mg cm^−2^, the battery maintained a capacity of 140.3 mAh g^−1^ with 97.2% retention after 3000 cycles, highlighting its excellent durability and strong potential for practical applications (Figure S23).

Movies S2 and S3 (Supporting Information) show that the ZiPy based Zn–I_2_ battery successfully light up an LED at −20 °C. After 45000 cycles at a high current density of 8 A g^−1^, the full battery with ZiPy electrolyte retained a capacity of 144.3 mAh g^−1^, with nearly 100% capacity retention and an average CE as high as 99.98%. The battery also exhibits excellent stability at −40 °C, further confirming its outstanding capacity retention capabilities (Figure 4h). Remarkably, even after 45 000 cycles at 8 A g^−1^ and −40 °C, the battery retains ≈90.1% of its initial capacity, proving its ability to operate stably at ultra‐low temperatures (Figures 4i; S24, Supporting Information). By comparing with the performances of previously reported Zn/I_2_ batteries, the ZiPy based Zn–I_2_ battery outperforms most counterparts in both capacity retention and low‐temperature endurance, highlighting its excellent promise for practical applications. (Figure 4j).^[^ ^8^, ^49^, ^50^, ^51^, ^52^, ^53^, ^54^ ^]^

## 3 Conclusion and Outlook

In summary, we describe an approach to utilize zwitterionic pyrrole (ZiPy) electrolyte additives to enhance the cycle life of Zn–I_2_ batteries. The improved Lewis base properties of pyrrole‐N group, along with the presence of quaternary ammonium cations, effectively mitigate the polyiodide shuttling effect, while sulfonic acid anions enhance the desolvation ability of Zn^2+^. The ZiPy additive significantly reduces zinc anode corrosion, suppresses the hydrogen evolution reaction (HER), and inhibits Zn dendrite growth, leading to improved cycle stability in Zn||Zn symmetric batteries, which can stabilize the cycling performance for 700 h at 5 mA cm^−2^ and 5 mAh cm^−2^. When being tested with an activated carbon‐supported iodine cathode, the Zn–I_2_ battery exhibited stable cycling over 45 000 cycles at a high current density of 8 A g^−1^. The ZiPy based Zn–I_2_ battery exhibits an excellent performance endurance at temperature as low as −40 °C. This facile and effective additive strategy holds great promise for practical applications in long‐life Zn–I_2_ batteries and shows great potential for operation under extreme conditions.

## Conflict of Interest

The authors declare no conflict of interest.

## Author Contributions

S.W. and Y.C. contributed equally to this work. S.W., S.Y.Z., B.B.X. and J.Y. conceived the idea for the manuscript and S.W. perform the synthesis of electrolyte. Y.C. performed the MD simulations and DFT calculates. S.W. and G.W. performed the electrochemical experiments. S.W., Y.C., S.D., Z.G., and E.A. analyse the data. S.Y.Z., B.B.X. and J.Y. drafted this manuscript. All authors discussed the results and edited the manuscript.

## Supporting information



Supporting Information

Supplementary Movie 1

Supplementary Movie 2

Supplementary Movie 3

## Data Availability

The data that support the findings of this study are available in the supplementary material of this article.
