# Supplementary material for: Zwitterionic Electrolyte Additives Empowered Robust Zn–I2 Batteries Enduring a Low Temperature of −40 °C
Source: Adv Sci (Weinh). 2025 Nov 6;13(5):e18135. doi: 10.1002/advs.202518135 (PMC12850064; doi:10.1002/advs.202518135)
Supplement: Supplementary file 1 — Supporting Information [file ADVS-13-e18135-s004.docx]

**Supporting Information for**

**Zwitterionic Electrolyte Additives Empowered Robust Zn-I_2_ Batteries Enduring a Low Temperature of -40 ℃**

Shuaibing Wang ^a ¶^, Yulong Chen ^a ¶^, Saddick Donkor ^b^, Zhanhu Guo ^b^, Gaopeng Wang ^a^, Yifan Li ^b^, Si Yu Zheng ^a^ *, Ben Bin Xu ^b^ *, Jintao Yang ^a^ *

S. Wang, Y. Chen, G. Wang, S. Zheng, Prof. J. Yang

^a^ College of Materials Science & Engineering, Zhejiang University of Technology, Hangzhou 310014, P. R. China

S. Donkor, Prof Z.Guo, Dr. Y. Li, Prof. B. B. Xu

^b^ School of Engineering, Physics and Mathematics, Faculty of Science and Environment, Northumbria University, Newcastle upon Tyne, NE1 8ST, UK

**Emails**: zhengsiyu@zjut.edu.cn (S. Zheng); Ben.xu@northumbria.ac.uk (B. B. Xu); yangjt@zjut.edu.cn (J. Yang)

^¶^ S. W. and Y. C. contributed equally to this work.

# Experimental Sections

# Methods

**Materials:** Zn(ClO_4_)_2_, Pyrrole, DMSO, THF, and Tetrabutylammonium bromide (TBAB) were purchased from Shanghai Aladdin Chemical Agent Co., Ltd. (China); 1,3-Propane sultone, and 3-(dimethylamino)propyl chloride hydrochloride were purchased from Sigma-Aldrich. Caron cloth, Carbon paper, Zinc foils (0.1 mm thickness), titanium foil (0.03 mm thickness), and Cu foil (0.05mm thickness) were purchased form Kelude Co., Ltd (China). All other chemicals were used as received.

**Preparation of zwitterionic pyrrole (ZiPy).** 0.05 mol of pyrrole is added to 20 mL of DMSO under a nitrogen atmosphere. Subsequently, 0.9 mmol of TBAB and 20 mL of a 50 wt% sodium hydroxide (NaOH) solution are added. The mixture is then stirred for 30 minutes. Following this, 0.05 mol of 3-(dimethylamino)propyl chloride hydrochloride is dissolved in 20 mL of DMSO and gradually introduced into the aforementioned mixed solution via dropwise addition. The reaction proceeds at room temperature for 24 hours. Subsequently, the reaction mixture is diluted with 50 mL of water to dissolve all salts. The product is then extracted with ether, and the combined organic layer is washed successively with 10 wt% aqueous NaOH solution, water and brine. The resulting solution is dried over magnesium sulfate (MgSO_4_), and the solvent is removed by vacuum evaporation to yield the intermediates. Finally, 170 mL of tetrahydrofuran (THF) is added, followed by the introduction of 0.04 mol of 1,3-propane sultone under a nitrogen atmosphere. The reaction is conducted at 50 °C for 48 hours to obtain the final product.

**Preparation of electrolytes.** 5 mol Zn(ClO_4_)_2_ was added into DI water to obtain 5 M Zn(ClO_4_)_2_ electrolyte. The modiated electrolytes were prepared by adding 0.12 mM, 0.24 mM and 0.36 mM ZiPy, respectively.

**Preparation of AC/I_2_ cathode.** Typically, 0.5 g of iodine powder was ground and mixed with 0.2 g of AC for 5 min. The mixture was sealed in a glass bottle and heated at 100 °C for 6 h. The cathode slurry was prepared by mixing AC/iodine (80 wt%), Super P (10 wt%) and CMC binder (10 wt %) in water to form a uniform slurry, which was stirred for 48 h. Then, the slurry was evenly coated on carbon paper using a doctor blade and dried at 50 °C for 6 h. The area iodine loading was controlled to be around 0.8~1.5 mg cm^-2^.

**Characterizations.** Fourier transform infrared (FT-IR) spectra were evaluated with a Nicolet 6700 FT-IR spectrometer. The crystal phases of the samples were tested by XRD with a Cu Ka irradiation source. The Raman mapping were recorded by LabRAM HR Evolution system (523 nm Ar laser). Confocal laser scanning microscope (CLSM) images were obtained by Olympus OLS4100. DSC was performed from -40 to 20 °C, with a heating rate of 10 °C/min and nitrogen flow flux of 10 mL/min.

**Electrochemical Measurements.** The Zn||Zn symmetric cells, Zn||Cu asymmetric cells, and Zn||I_2_ full cells were assembled in CR2032 coin cells. A glass fiber (GF/D) was selected as the separator, and the added electrolyte was 120 µL. The electrochemical performance of all the cells were tested by the battery test system (Neware CT-4008T). Cyclic voltammetry (CV), Tafel plots, and electrochemical impedance spectroscopy (EIS) measurements were performed on a CHI 760E electrochemical workstation.

**Density functional theory calculations.** The interaction between ZiPy, Zn^2+^, and water molecules was calculated using Density Functional Theory (DFT) with the ORCA program1. The r2SCAN-3c functional was used for structure optimization. The binding energy between the monomers and the salt ions and water molecules was defined as follows:

$E_{B}=E_{S}-E_{1}{-E}_{2}$……….. (1)

Where $E_{S}$ is the total energy of whole system, $E_{1}$ is the total energy of component 1, $E_{2}$is the total energy of component 2.

All other quantum chemical calculations, including geometric optimization and frequency calculations, are carried out at the b3lyp/lanl2dz level. Dispersion corrections with the DFT-D3 method incorporating BJ damping were implemented to address weak intermolecular interactions and enhance-computational accuracy. The above calculations were conducted using Gaussian 16Wsoftware.The binding energy ($\Delta_{E}$) was calculated as follows:

$\Delta_{E}=E_{complex}-E_{a}{-E}_{b}$……….. (2)

where the $E_{complex}$is the energy of the optimized system, $E_{a}$ is the energy of Zipy molecule (or Zn^2+^ions); $E_{b}$is the energyof·the optimized·I_3_^-^ and I_5_^-^.

**Molecular dynamics (MD) simulations.** The simulation system of Zn(ClO_4_)_2_ electrolyte contains 2690 H_2_O molecules and 200 Zn(ClO_4_)_2_ units (corresponding to the experimental system of 5 M Zn(ClO_4_)_2_ electrolyte), while the modified electrolyte contains additional 18 ZiPy molecules (that with 0.24 mM ZiPy). The OPLS-AA force field was employed for Zn(ClO_4_)_2_ and ZiPy. However, for H_2_O molecules, the SPC water model was adopted. Long-range Coulombic interactions were calculated using the particle-particle particle-mesh (PPPM) solver. Initial relaxation was performed under the isothermal-isobaric (NPT) ensemble for 20 ns with a time step of 0.5 fs. After equilibration, a 10 ns production run was conducted under the canonical (NVT) ensemble. Temperature at 298.15 K (room temperature) and pressure at 1 atm (1.01 × 10^5^ Pa) were regulated using the Nosé-Hoover thermostat and barostat, with damping parameters set to 0.1 ps and 1 ps, respectively. Periodic boundary conditions were applied to all three dimensions of the simulation box. The equations of motion were integrated using the velocity-Verlet algorithm. All MD simulations were carried out using the open-source LAMMPS package.

# Supporting Figures


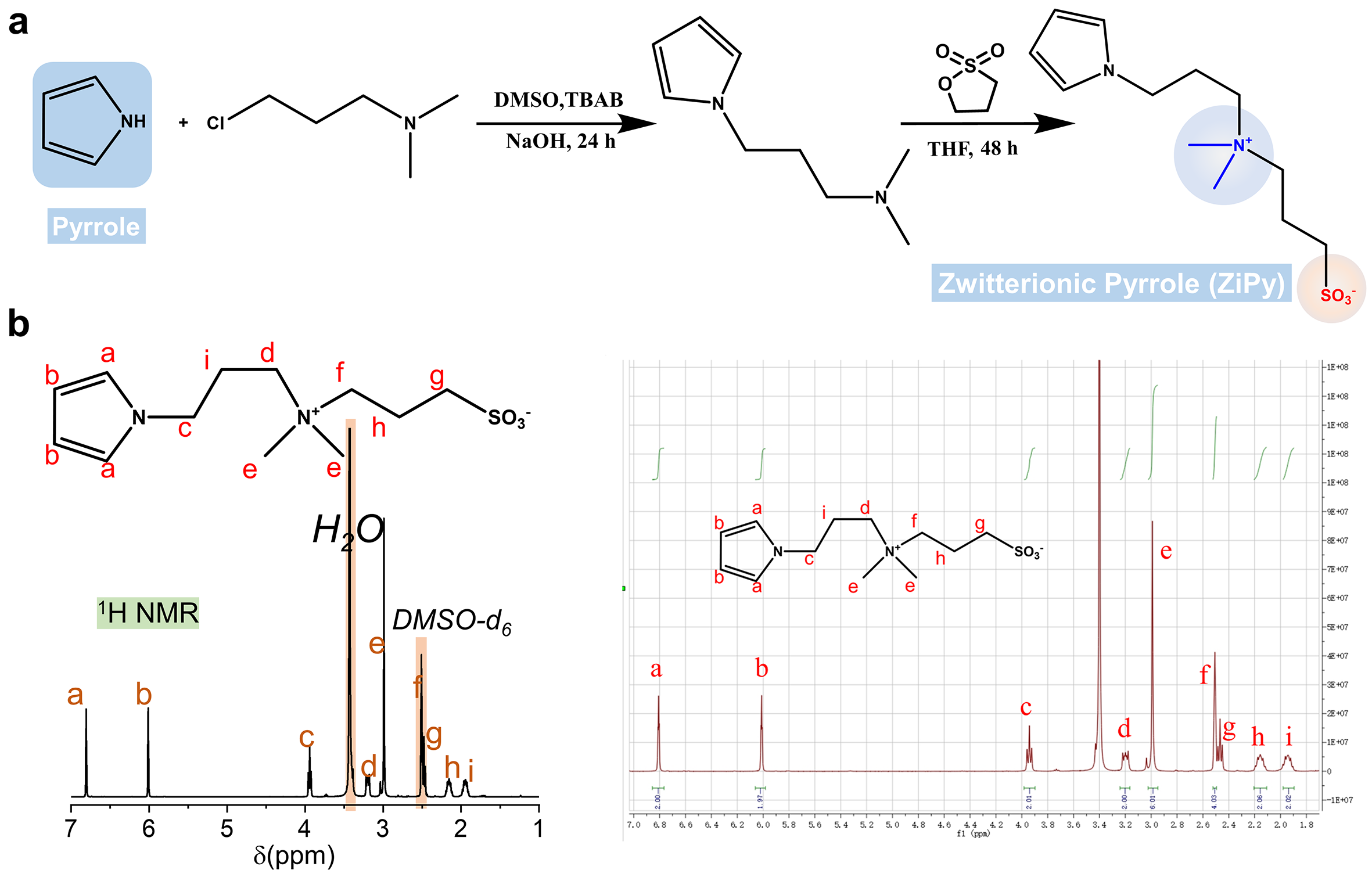


**Fig. S1.** (a) The synthesis process of ZiPy and, (b) corresponding ^1^H nuclear magnetic resonance spectrum (DMSO-d_6_).


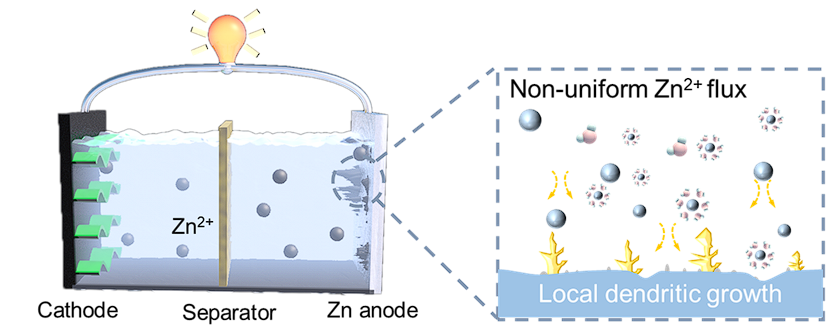


**Fig. S2.** Schematic diagram of uneven Zn^2+^ deposition in the control group.


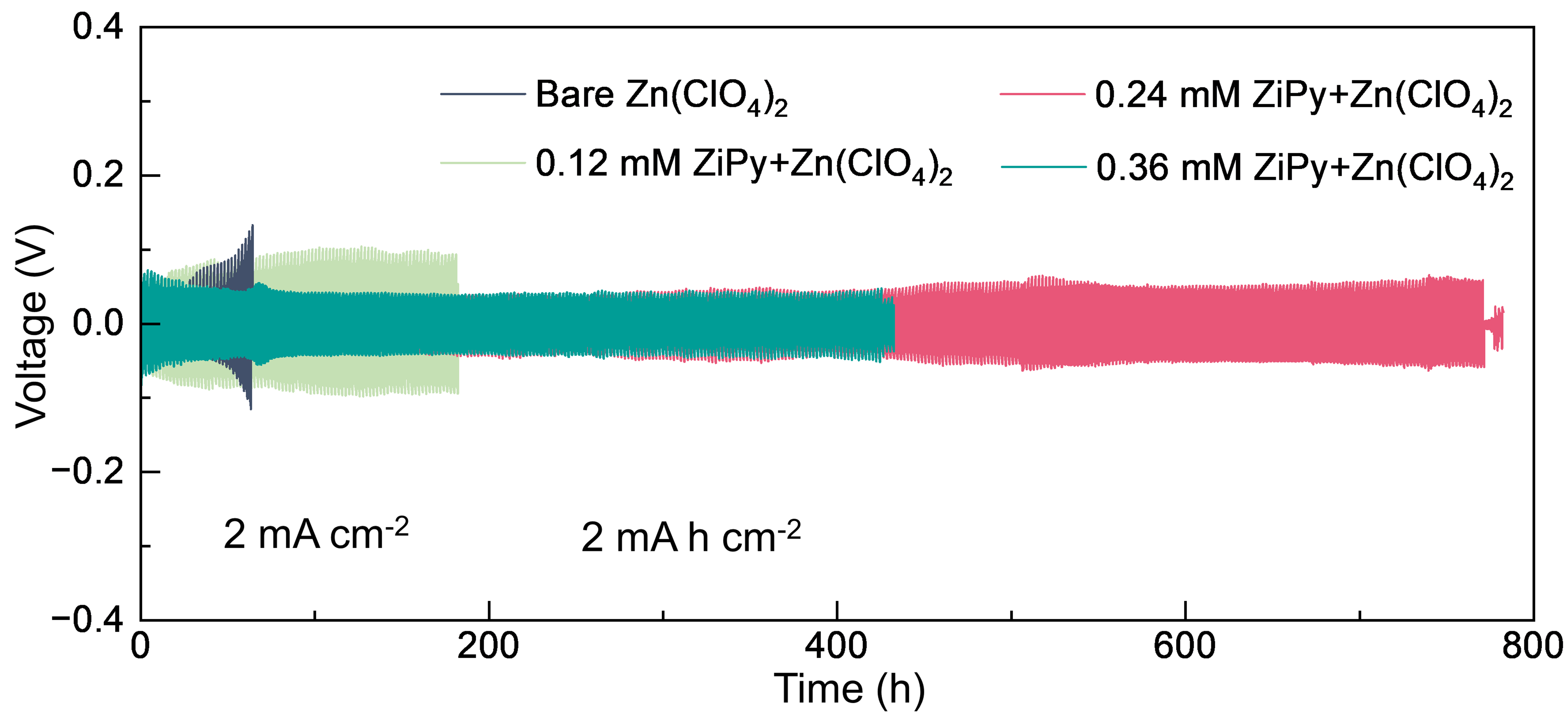


**Fig. S3.** Cyclic deposition curves of zinc symmetric cells in electrolytes with various ZiPy concentrations under 2 mA cm^-2^ and 2 mAh cm^-2^.


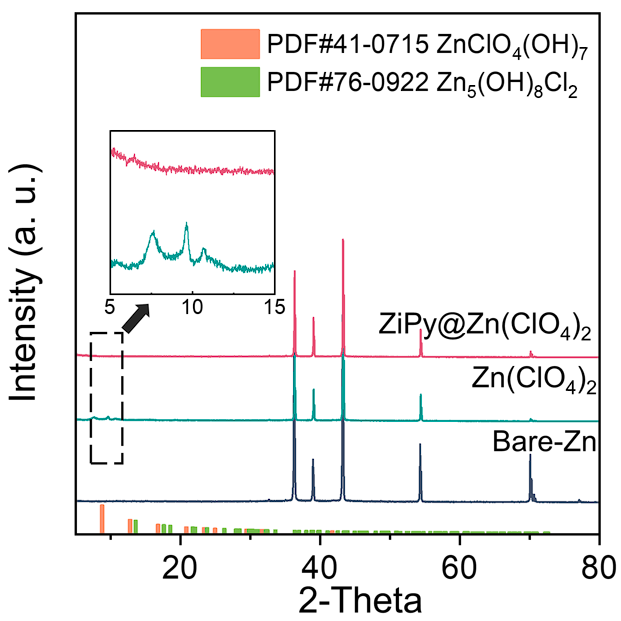


**Fig. S4.** XRD results after immersion in different electrolytes for 7 days.


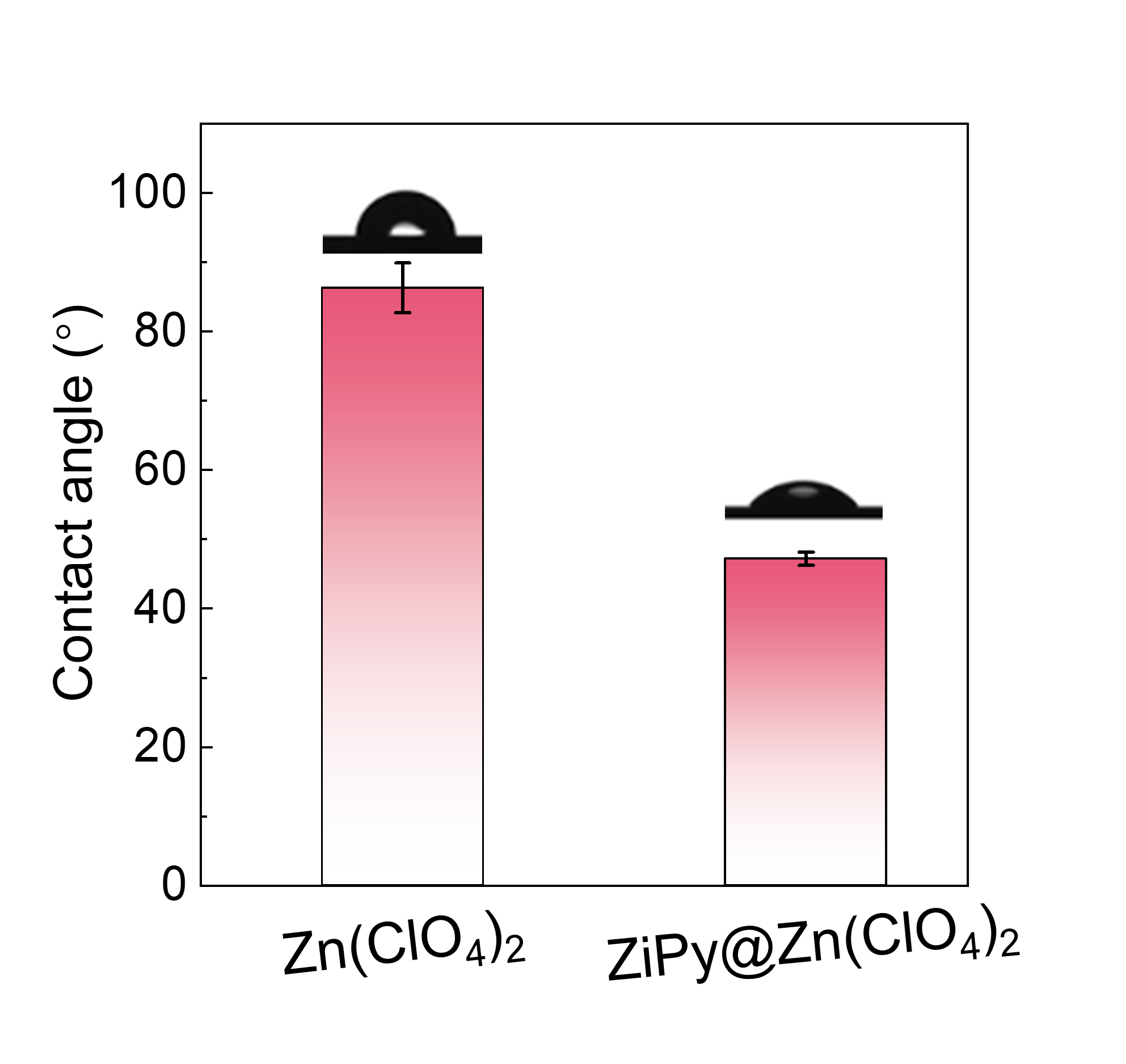


Fig. S5. Contact angle of zinc anode under different electrolytes.


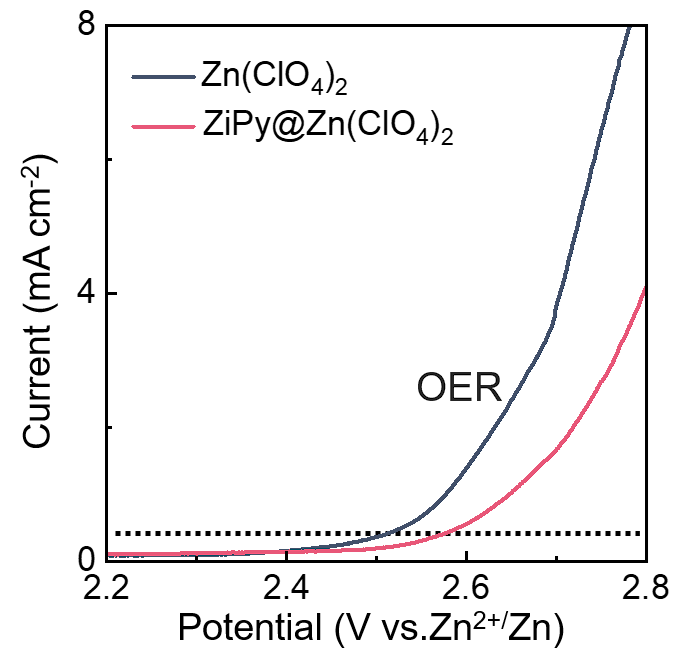


**Fig. S6.** The LSV tests to evaluate the OER performance.


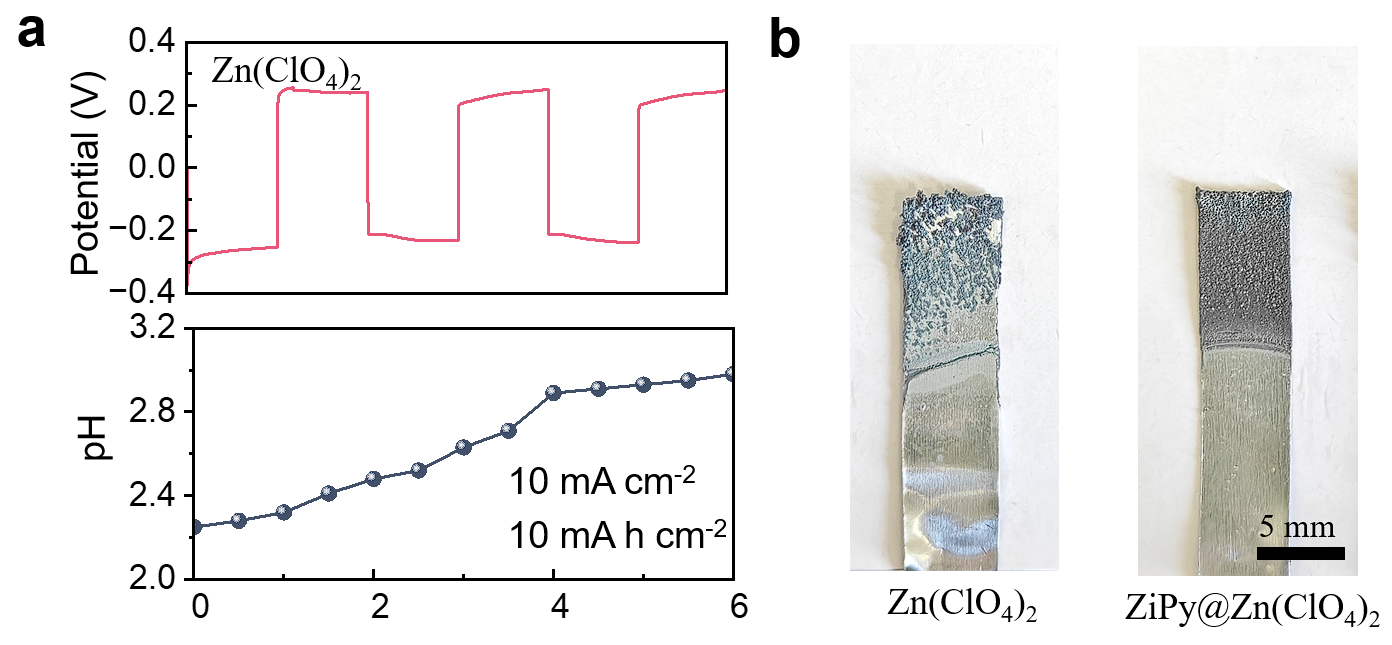


**Fig. S7.** (a) pH monitoring of the Zn(ClO_4_)_2_ electrolytes of Zn//Zn symmetric cells cycling at 10 mA cm^-2^, and (b) corresponding electrode images.

**
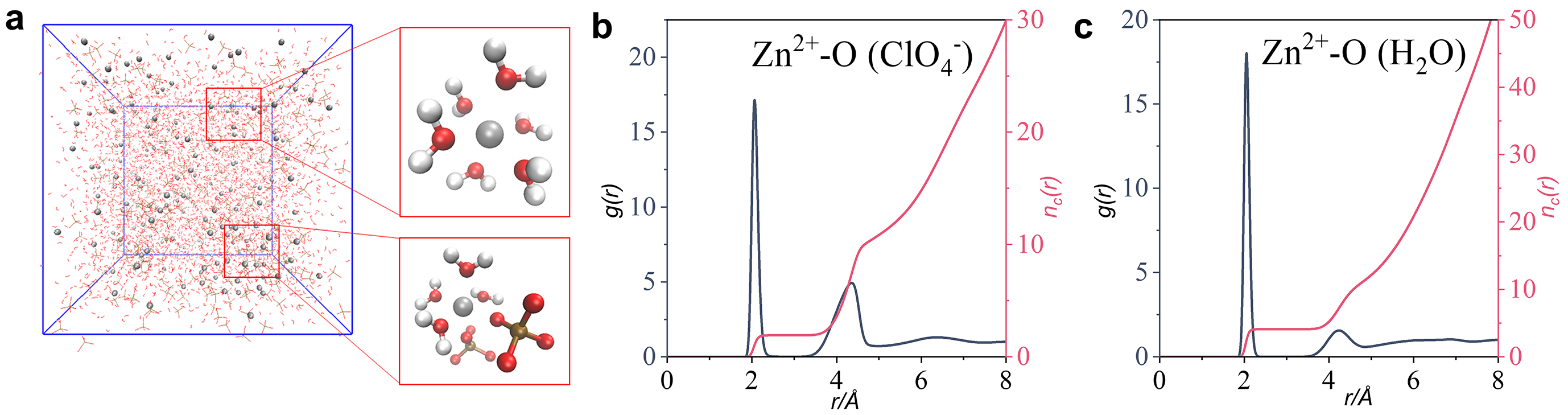
**

**Fig. S8.** (a) 3D snapshot for a simulation system of Zn(ClO_4_)_2_ electrolyte and the corresponding solvation structures. (b) RDF (g(*r*), left axis) and coordination number (*n*_c_(*r*), right axis) of O atoms in ClO_4_^-^ and (c) that in H_2_O from a referenced Zn^2+^ obtained from MD simulation.


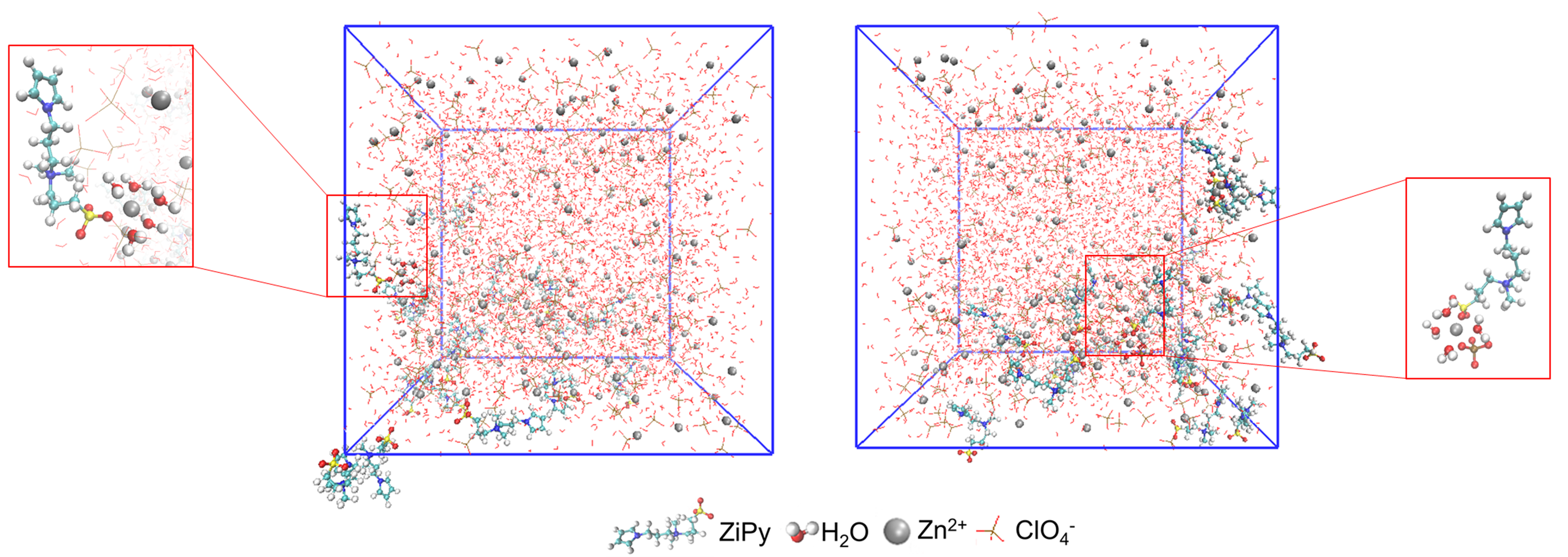


**Fig. S9**. 3D snapshot of the ZiPy-containing additive system.


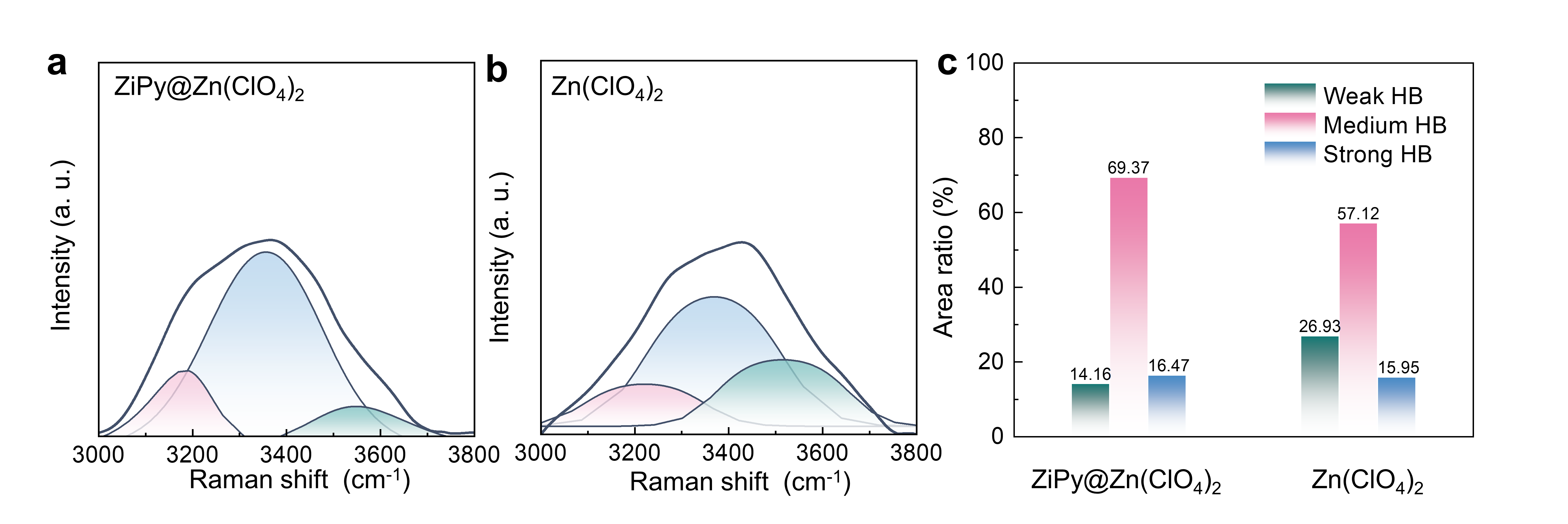


Fig. S10. Raman spectra of (a) ZiPy@Zn(ClO_4_)_2_ and (b) Zn(ClO_4_)_2_ electrolytes. (c) The calculated ratio of various H-bonds in different electrolytes from fitted curves.


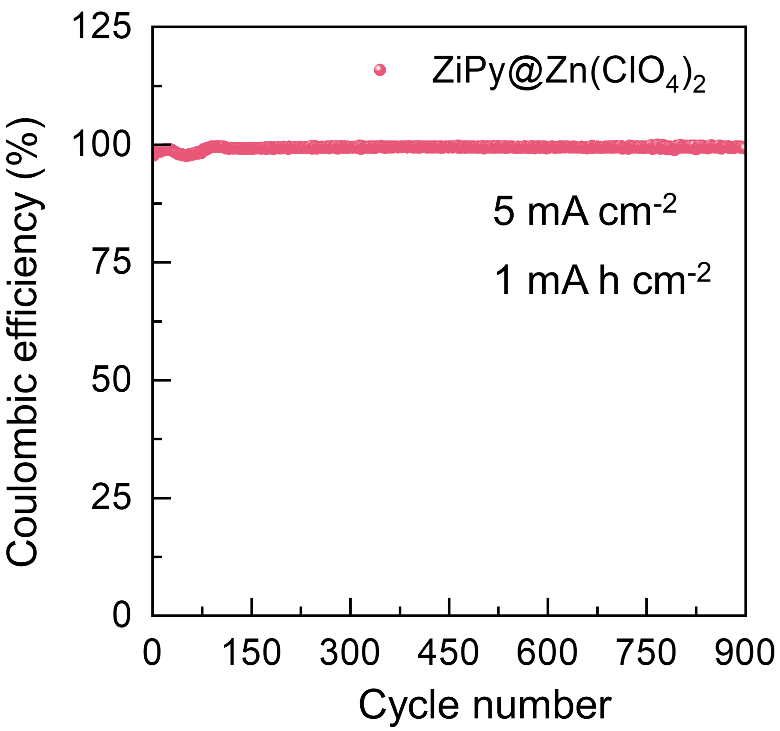


**Fig. S11.** CE of Zn plating/stripping on Cu substrate.


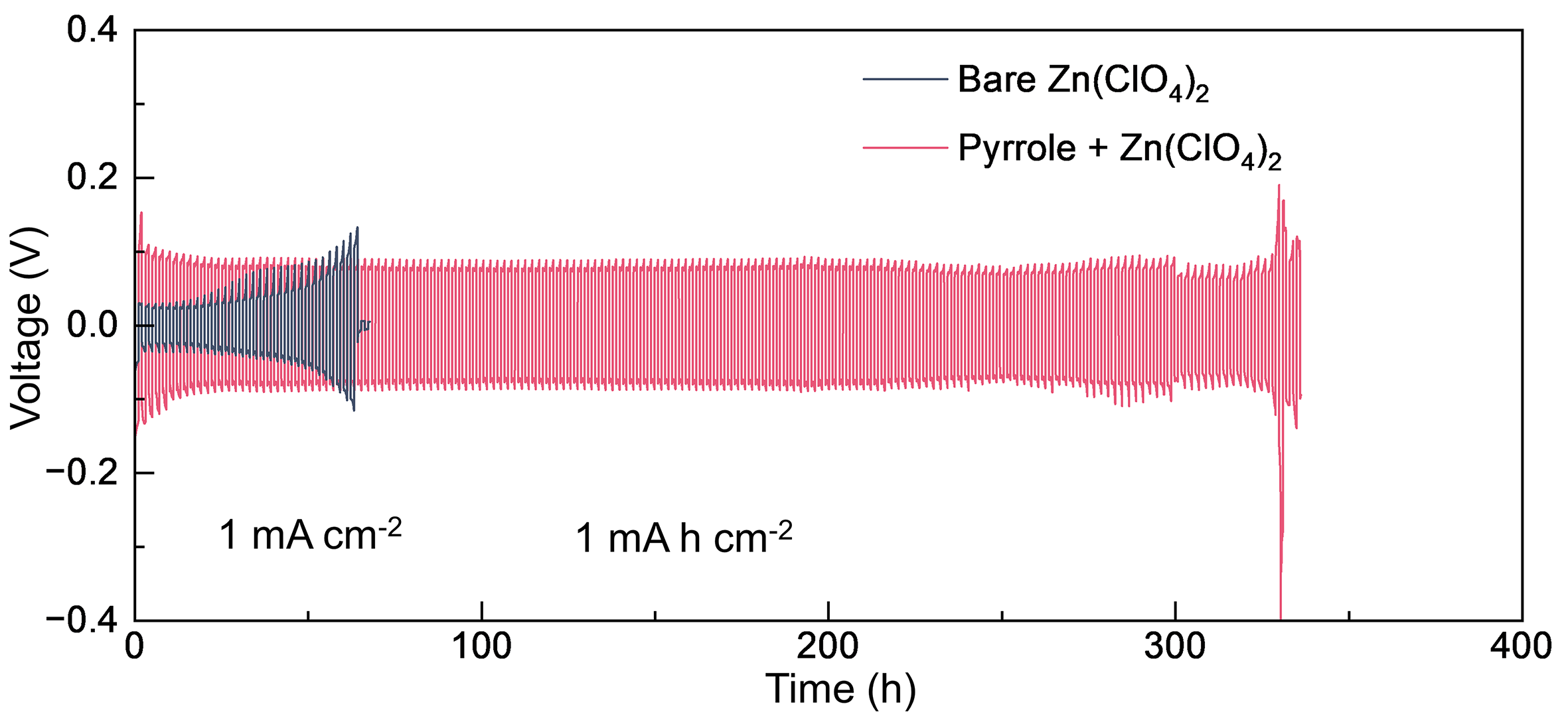


**Fig. S12.** Comparison of cycling performance of symmetric batteries after adding pyrrole.


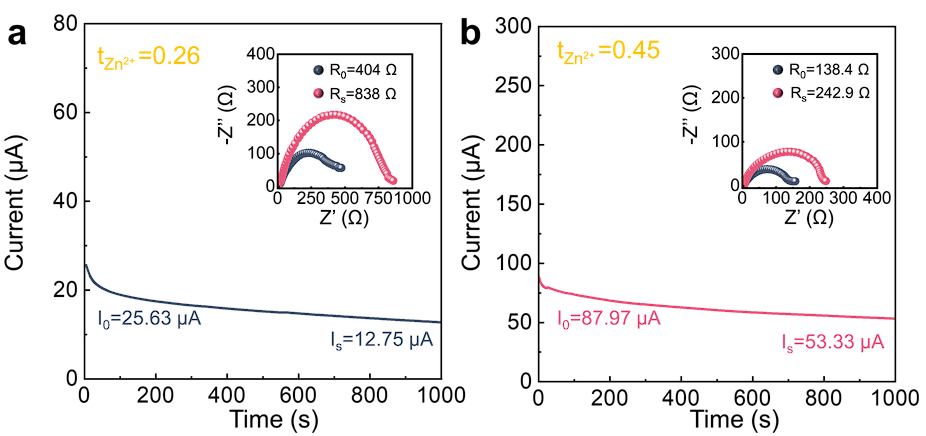


Fig. S13. Current-time curves of the Zn/Zn symmetric batteries using (a) Zn(ClO_4_)_2_ and (b) ZiPy@Zn(ClO_4_)_2_ electrolytes. The inset shows the EIS changes before and after polarization.


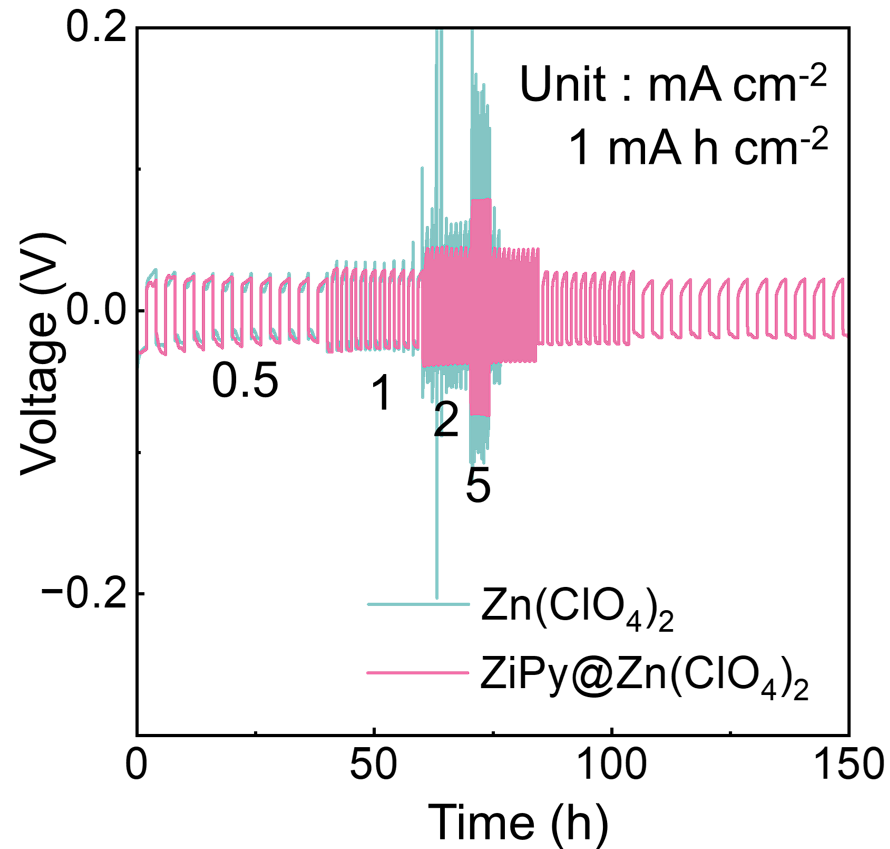


**Fig. S14.** Cyclic deposition curves of zinc symmetric cells under various current densities.


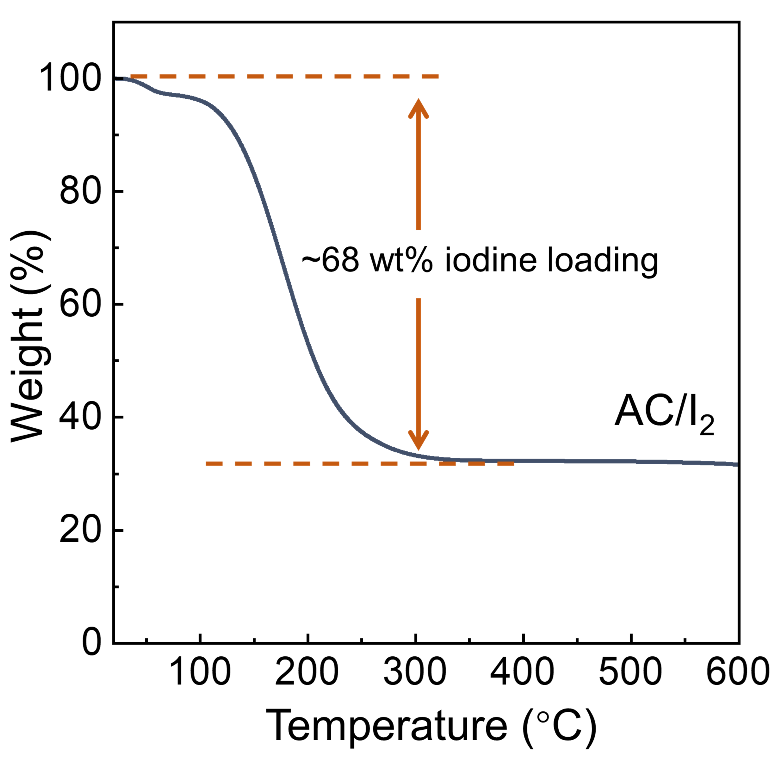


**Fig. S15.** TG curves of AC/I_2_ under the temperature of 25 to 600 °C.


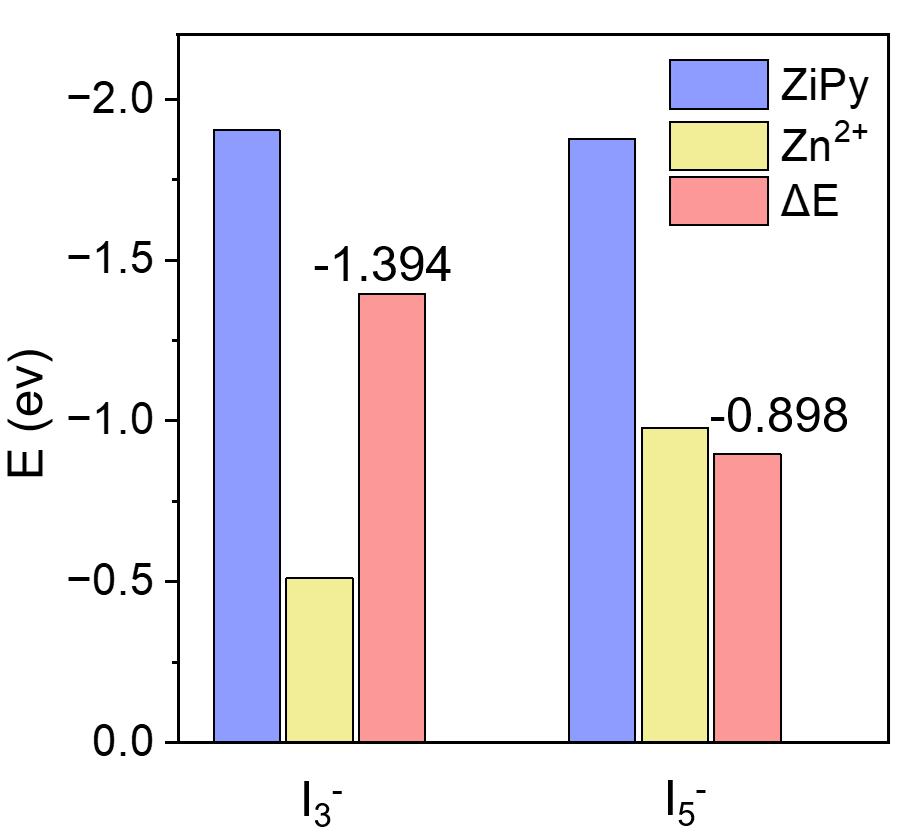


**Fig. S16.** The adsorption energy of polyiodides on Zn^2+^ and ZiPy.


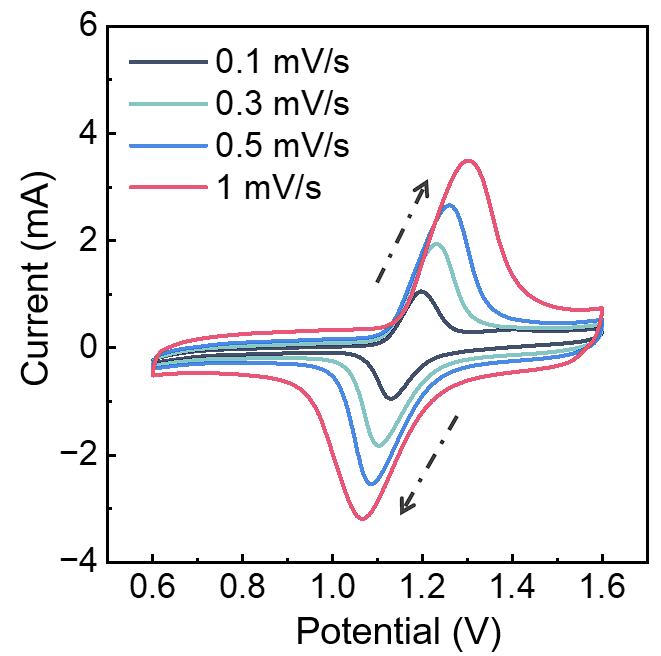


**Fig. S17.** CV curves of Zn-I_2_ battery containing ZiPy electrolyte at different scan rates.

**
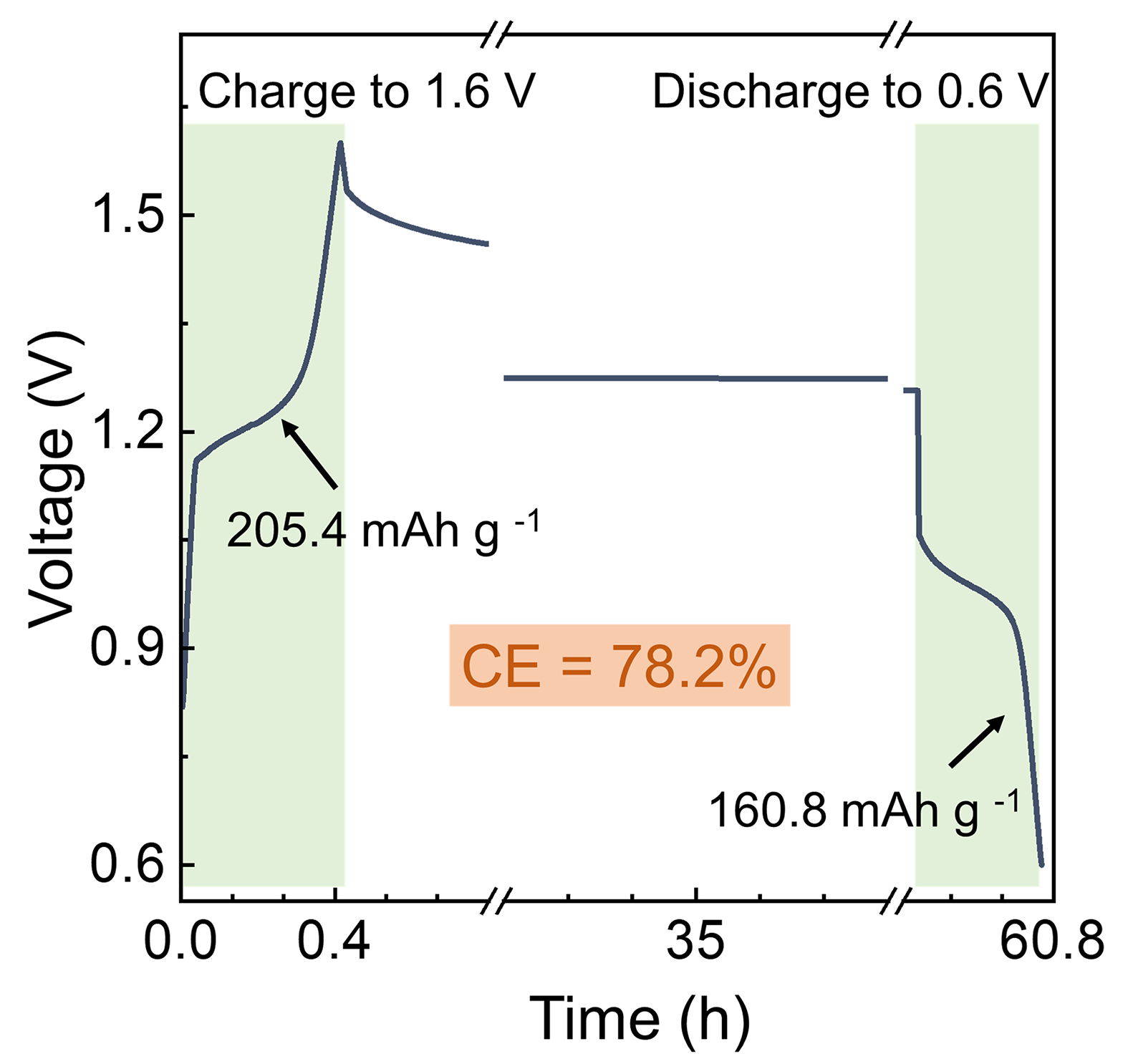
**

**Fig. S18.** Capacity retention of Zn-I_2_ battery in Zn(ClO_4_)_2_ electrolyte after 60 h.

**
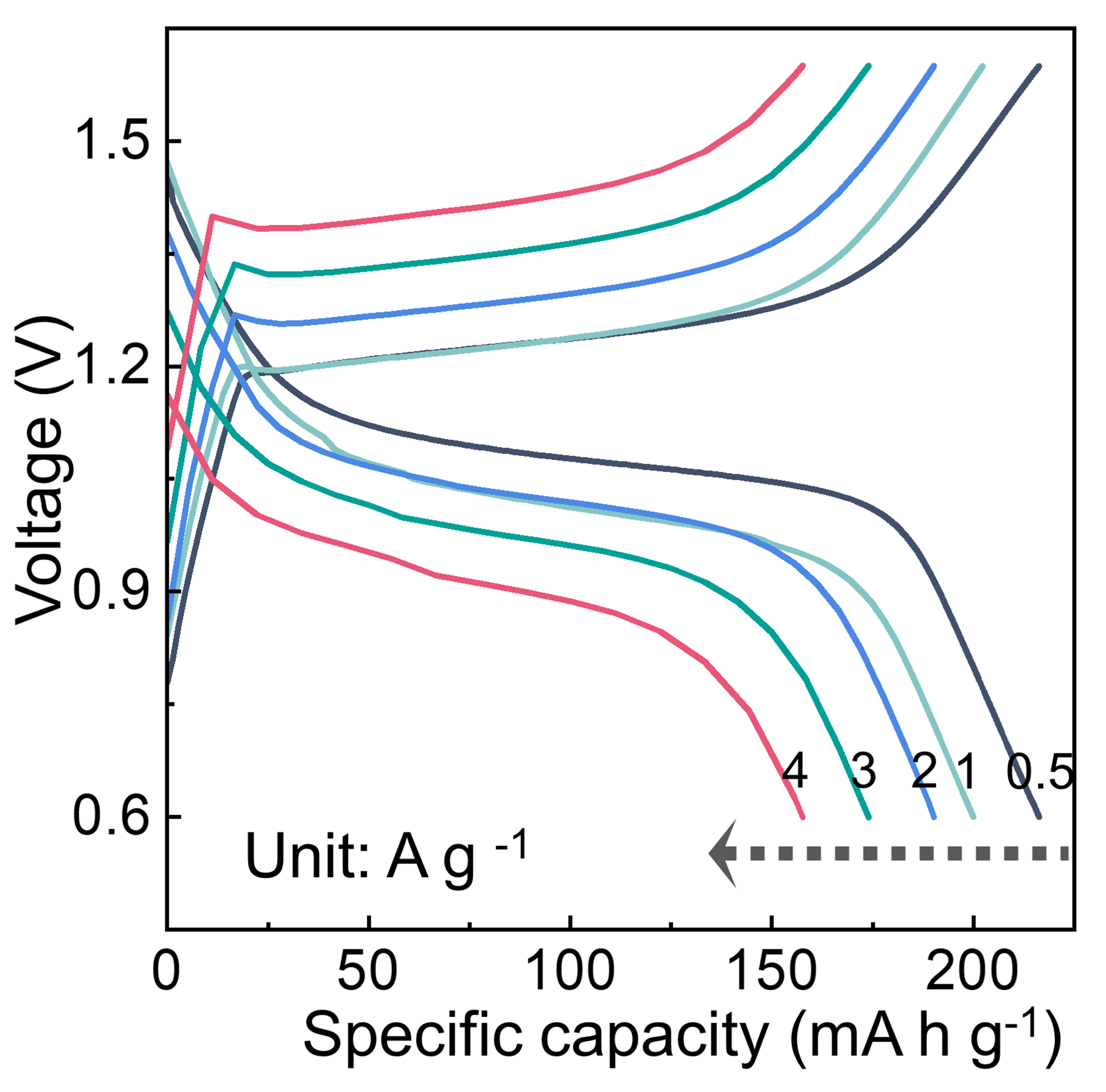
**

**Fig. S19.** GCD curves of Zn-I_2_ battery in ZiPy@Zn(ClO_4_)_2_ electrolyte at varied current densities.

**
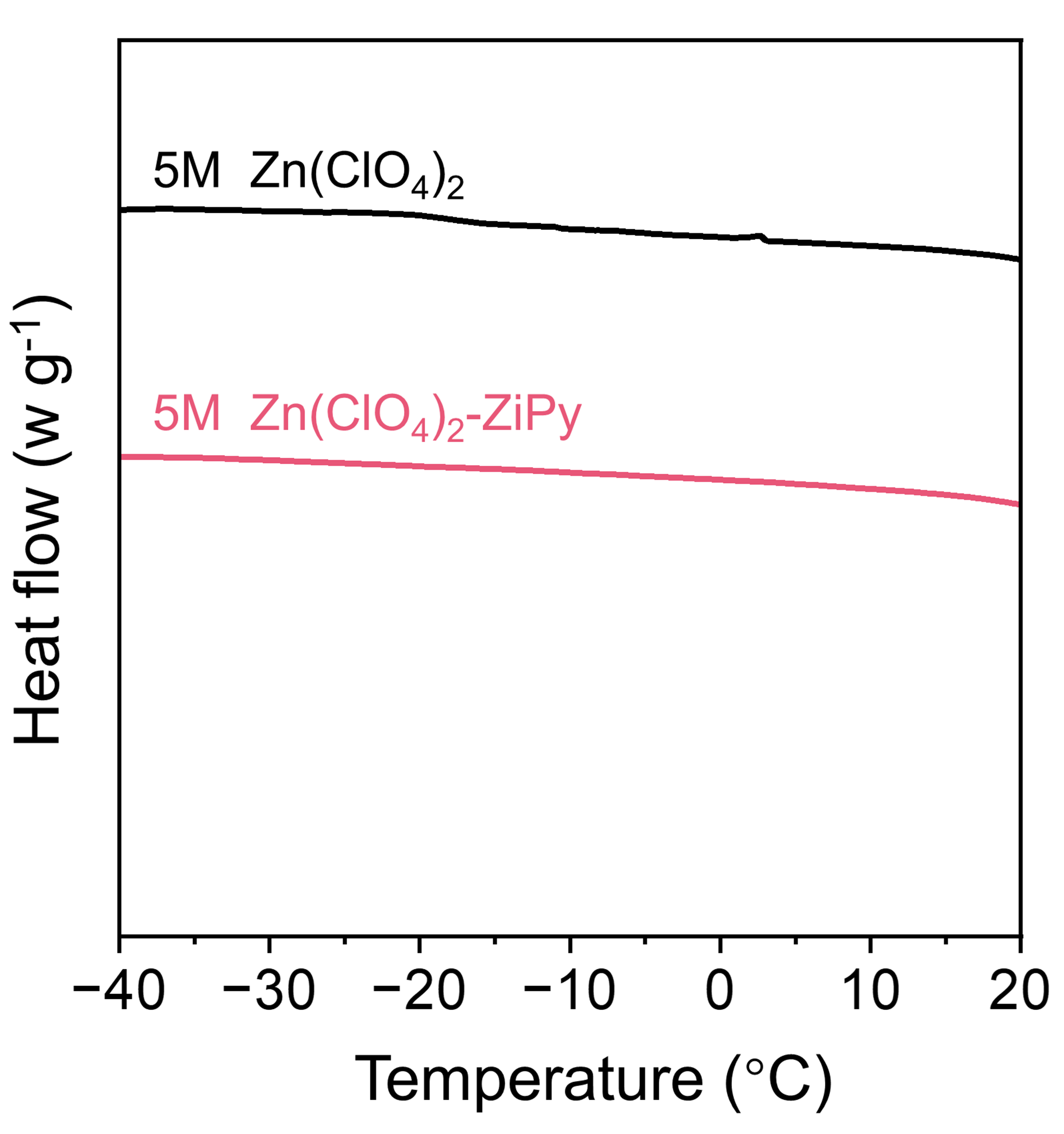
**

**Fig. S20.** DSC curves of different electrolytes at -40 to 20 °C.
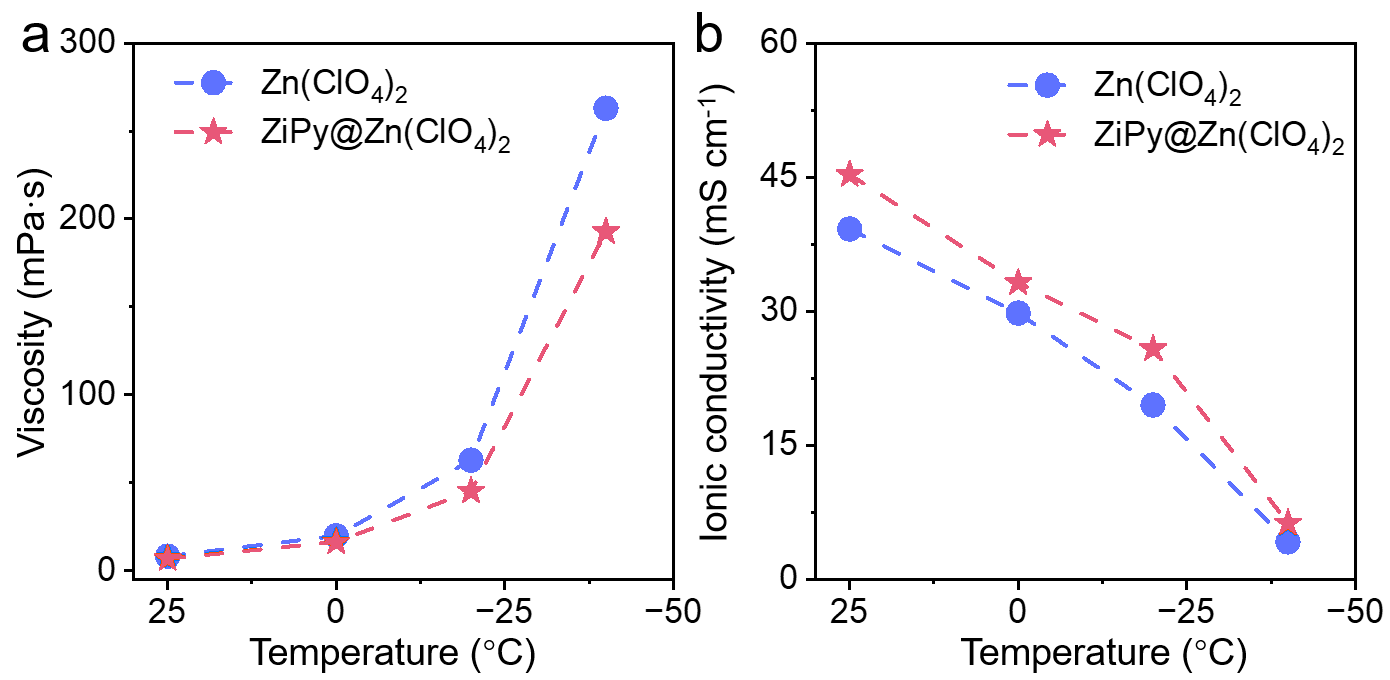


**Fig. S21.** The viscosity and ionic conductivity of different electrolytes at varied temperatures.


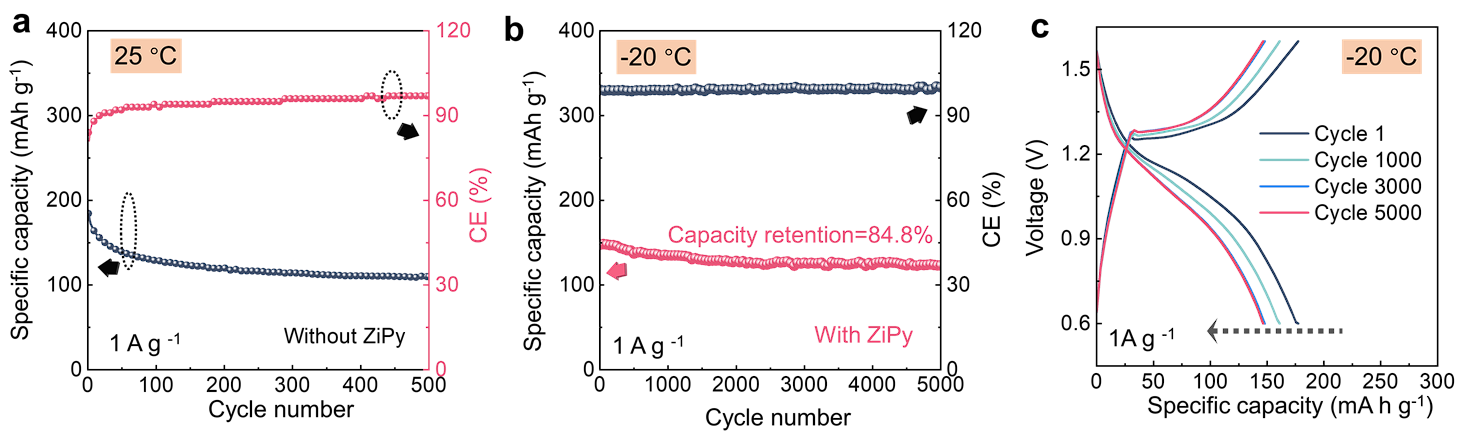


**Fig. S22.** (a) Capacity retention and CE of Zn-I_2_ full battery at 25 °C. (b) Capacity retention and CE of Zn-I_2_ full battery at -20 °C and (c) GCD curves with different cycle times.


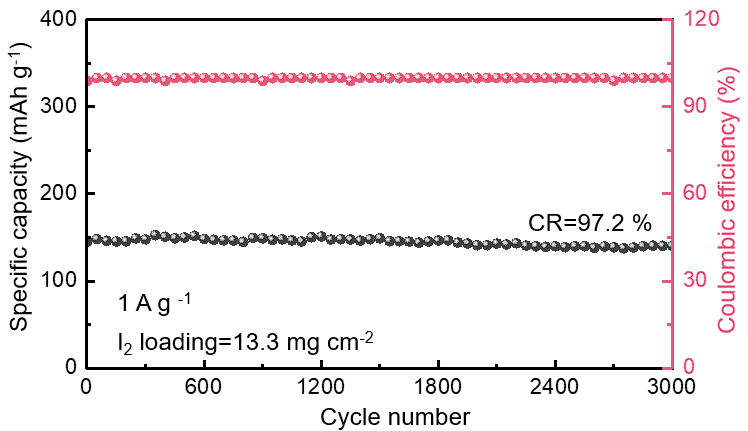


**Fig. S23.** The cycling stability of Zn-I_2_ battery at high I_2_ loading of 13.3 mg cm^-2^.


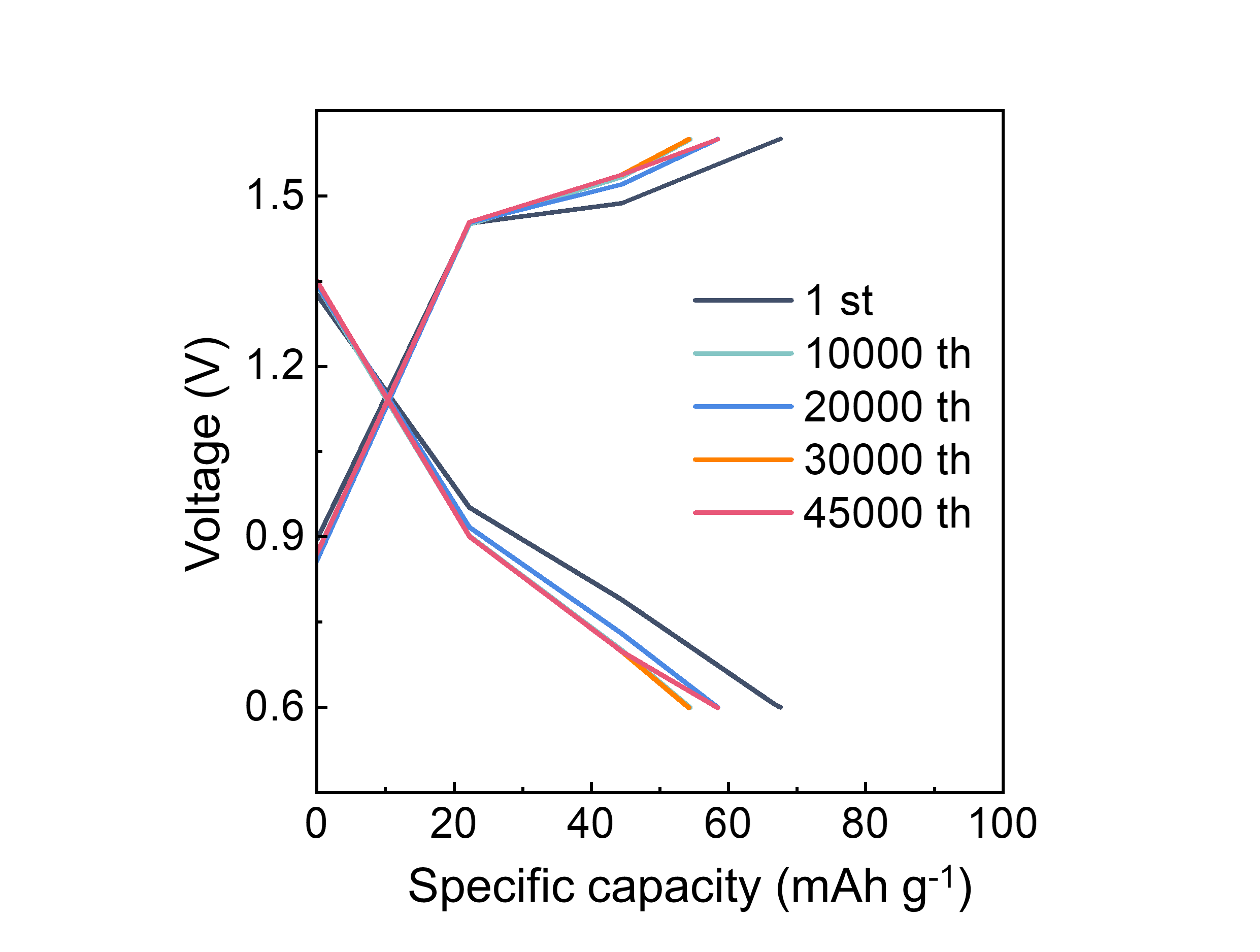


**Fig. S24.** The GCD curves of the Zn-I₂ battery at -40 °C for different cycle numbers.
